# Synthesis of New Ruthenium Complexes and Their Exploratory Study as Polymer Hybrid Composites in Organic Electronics

**DOI:** 10.3390/polym16101338

**Published:** 2024-05-09

**Authors:** Ricardo Ballinas-Indilí, María Elena Sánchez Vergara, Saulo C. Rosales-Amezcua, Joaquín André Hernández Méndez, Byron López-Mayorga, René Miranda-Ruvalcaba, Cecilio Álvarez-Toledano

**Affiliations:** 1 Departamento de Ciencias Químicas, Facultad de Estudios Superiores Cuautitlán Campo 1, Universidad Nacional Autónoma de México, Avenida 1o de Mayo s/n, Colonia Santa María las Torres, Cuautitlán Izcalli 54740, Mexicomirruv@yahoo.com.mx (R.M.-R.); 2 Facultad de Ingeniería, Universidad Anáhuac México, Av. Universidad Anáhuac 46, Col. Lomas Anáhuac, Huixquilucan 52786, Mexico; 3 Instituto de Química, Universidad Nacional Autónoma de México, Circuito Exterior s/n, Ciudad Universitaria, México City 04510, Mexicocecilio@unam.mx (C.Á.-T.); 4 Escuela de Química, Facultad de Ciencias Químicas y Farmacia, Universidad de San Carlos de Guatemala, 11 avenida, Ciudad de Guatemala 01012, Guatemala; byron.lopez@profesor.usac.edu.gt

**Keywords:** indanone, ruthenium complexes, poly(methyl methacrylate), polymer hybrid composite, semiconductor film, bandgap

## Abstract

Polymeric hybrid films, for their application in organic electronics, were produced from new ruthenium indanones in poly(methyl methacrylate) (PMMA) by the drop-casting procedure. Initially, the synthesis and structural characterization of the ruthenium complexes were performed, and subsequently, their properties as a potential semiconductor material were explored. Hence hybrid films in ruthenium complexes were deposited using PMMA as a polymeric matrix. The hybrid films were characterized by infrared spectrophotometry and atomic force microscopy. The obtained results confirmed that the presence of the ruthenium complexes enhanced the mechanical properties in addition to increasing the transmittance, favoring the determination of their optical parameters. Both hybrid films exhibited a maximum stress around 10.5 MPa and a Knoop hardness between 2.1 and 18.4. Regarding the optical parameters, the maximum transparency was obtained at wavelengths greater than 590 nm, the optical band gap was in the range of 1.73–2.24 eV, while the Tauc band gap was in the range of 1.68–2.17 eV, and the Urbach energy was between 0.29 and 0.50 eV. Consequently, the above comments are indicative of an adequate semiconductor behavior; hence, the target polymeric hybrid films must be welcomed as convenient candidates as active layers or transparent electrodes in organic electronics.

## 1. Introduction

The mechanical flexibility of polymers is attractive for the possibility to have stable film electronics performance on non-flat surfaces [1]. This conformable electronics is achieved by thin, lightweight, and transparent polymers [1,2] as the polyimide [1,3,4,5], the polyacrylamide [6], the polyester [7], the polyethylene napthalate [8], or the polymethyl methacrylate [9,10]. Polymethylmethacrylate (PMMA) has been highlighted for its use in electronic devices and as an optical component because of its volume productivity and low cost [11]. PMMA is a flexible and transparent polymer that can act as a dielectric layer [10], substrate [1,9], and resistor layer [12] in conformable electronics. However, PMMA has poor chemical resistance, a low melting point, and low electrical properties [11], which can make its use difficult in devices in corrosive or high temperature environments. To solve the issues mentioned above, its properties can be modified by the formation of hybrid films with a polymer as the matrix and some small molecules with semiconductor behavior as the indanones.

**Figure 1:**
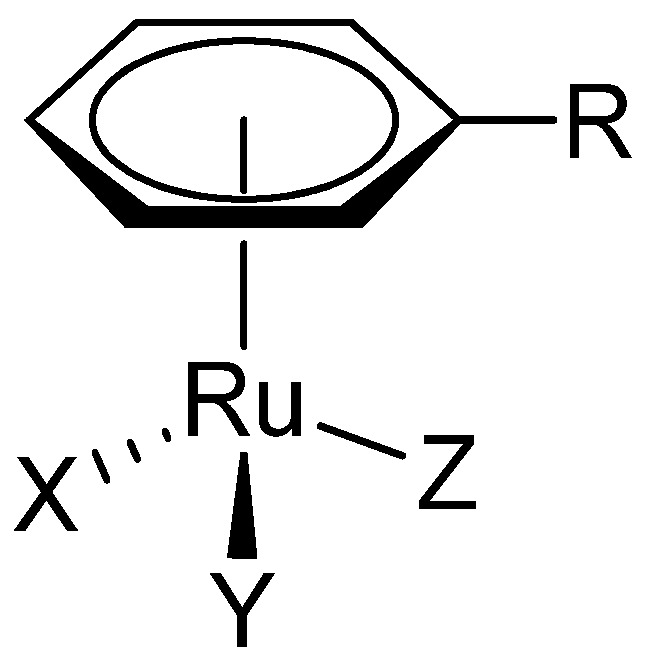
Half-sandwich or piano-stool structure of ruthenium complex.

The use of indanones with different metals, such as iron, copper, tin, vanadium, and uranium, to increase their semiconductivity has also been reported by some authors in this work [13,14,15,16,17]. The complexes derived from 2-benzylidene-1-indanones are stable semiconductors with interesting optical and electrical properties due to their π-conjugate system and the presence of transition metals in their structure. Furthermore, due to their high chemical and thermal stability, hybrid films can be fabricated with these complexes. It has been found that these films have the potential to be used as active layers in the production of optoelectronic devices [13,14,15,16,17], however, the synthesis and semiconductor properties of ruthenium complexes with these indanones as ligands have not yet been. There are few reports on ruthenium compounds embedded in polymer matrices for the study of prolonged release of small molecules [18], chemical vapor deposition for the exploration of potential properties in materials [19], as well as their evaluation in non-linear optics applications [20]. In recent years, ruthenium (Ru) has gained great interest in the design of bioactive molecules and catalysts, due to its affinity with an important diversity of monodentate and/or polydentate ligands. Ru is a transition metal in the second series, whose most common oxidation states are II (d6) and III (d5). Ru is a Lewis acid, with a great affinity for “soft” ligands, such as R_3_P, R_2_S, CN^−^, NO_2_^−^, alkenes and alkynes, and little affinity for “hard” ligands, such as O, F, and Cl. There are a great variety of Ru(II) compounds that contain in their structure different ligands with very varied characteristics, both structural and electronic, that can be coordinated to give rise to organometallic compounds. Within these complexes are the arene–ruthenium types, where ruthenium presents a coordination environment in which three sites are occupied by an arene attached to a ring, while several ligands can occupy the other three coordination sites, whose bonds, metal–carbon (Ru–C), can donate six electrons and are highly stable in acidic, basic, reducing, or oxidizing conditions. Complexes with this geometry are known as half-sandwich or piano-stool. Figure 1 shows the reactivity sites that give rise to a variety of structural chemical designs in which the Ar substituent, the monodentate ligands, Y and Z, or the bidentate ligand XY modulate the chemical, physical, and electronic properties. The function played by the arene ligand is to stabilize the charge of the metal to Ru(II), modulating the hydrophobicity when functionalized; in addition, it influences the lability of the monodentate ligand X by varying the degree of π donation. The Z ligand, usually a halogen, modulates the coordination rate of Ru towards reactive molecules. Also, the ligands X and Y influence the activity and mode of action of the complex. Due to this versatility, these molecules are used in catalysis [21], supramolecular chemistry [22,23], and solar cells [24].

Based on the mentioned characteristics of PMMA and ruthenium indanones, in this work in a first stage, is offered the preparation of a set of PMMA-Ru indanone hybrid films. In addition, it is expected to combine the semiconductor properties of ruthenium complexes with the mechanical properties of PMMA to obtain films with band gap in the range of organic semiconductors used in optoelectronics. This work also includes the deposition of polymer hybrid films in new piano-stool ruthenium complexes using hydroxy benzylidene indanones as a ligand. This type of piano-stool ruthenium complexes has almost not been explored for their properties as semiconductors. Some works have focused on the study of ruthenium complexes, however, only with a nonlinear optics (NLO) approach. With the objective of determining whether, thanks to their properties, ruthenium complexes have a semiconductor character that allows them to be used in flexible organic light-emitting diodes. The use of organic diodes with polymer hybrid films can have certain advantages over other kinds of diodes such as a greater sensitivity, a greater mechanical resistance, and the elimination of an external voltage [25,26]. Hence, the goal of this work is related to the synthesis of five new ruthenium complexes and the development of new polymer hybrid composites that can be used in an emerging category of organic semiconductor films.

## 2. Materials and Methods

The following reagents were used as received: *o*-phthalaldehyde, acetophenone, 4′-methoxiacetophenone, 4′-chloroacetophenone, 4′-bromoacetophenone, 4′-iodoacetophenone, dichloro (*p*-cymene) ruthenium (II) dimer ([Cy-Ru]), dry dichloromethane, ethyl acetate, *n*-hexane, ethanol, methanol, and silica gel 60 (0.063–0.200 mm, 70–230 mesh ASTM, acquired from Merck–Millipore, Steinheim, Germany). The melting points were obtained using a Melt-Temp II apparatus and are uncorrected. FTIR spectrophotometry was obtained using Bruker Tensor 27 equipment (Bruker, Ettlingen, Germany). The acquisition of ^1^H and ^13^C NMR spectra were obtained in CDCl_3_ using Bruker 500 Ascend equipment (Bruker, Ettlingen, Germany) at 400 and 100 MHz, respectively. The mass spectra were obtained with JEOL-JMX-X-103 equipment (JEOL, Tokyo, Japan). The FAB+ ionization method used a polyethylene glycol 600 matrix and a high-resolution mass measurement was achieved using an Agilent Tech spectrometer (Agilent Technologies, Santa Clara, CA, USA). The HRMS-DART+ (19 eV) spectra were obtained with a JEOL JMS-T100LC spectrometer (JEOL, Tokyo, Japan) and a HPLC-EM-SQ-TOF, Model G6530BA, Agilent Tech (Agilent Technologies, CA, USA).

### 2.1. Synthesis of Ligands **1a**–**e**

**Scheme 1:**
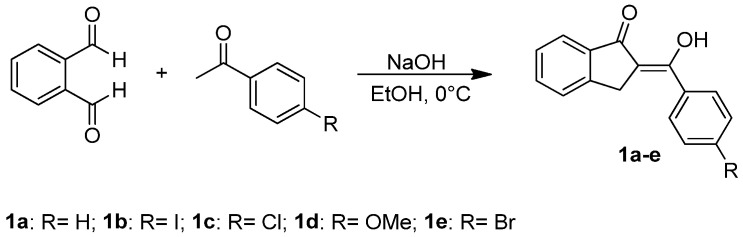
Indanones general reaction **1a**–**e**.

The synthesis of hydroxymethilidene indanones (Scheme 1) was carried out based on a procedure previously reported by our research group [17]. *o*-phtalaldehyde was added to an ethanolic solution of sodium hydroxide (2.5 eq) with the corresponding acetophenone (1.0 eq). The mixture was stirred at 0 °C and then poured over ice and hydrochloric acid until a pH of 3 was reached. The resulting solid was filtered and purified by column chromatography, using eluent ethyl acetate/*n*-hexane in a polarity gradient.

**Figure 2:**
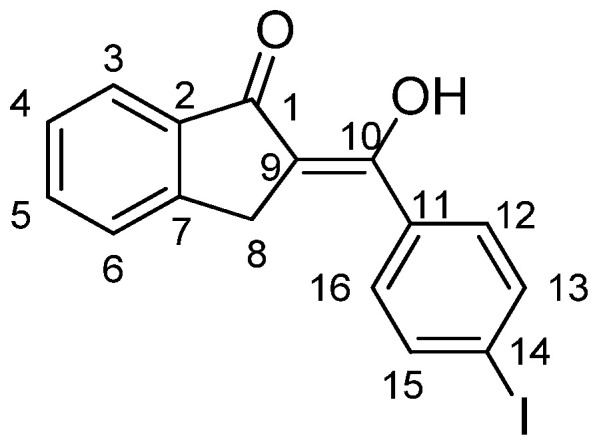
Structure of indanone **1b**.

It is important to note that ligand **1b** (Figure 2) is a new molecule; consequently, the physical and spectroscopic information is incorporated in the manuscript. The rest of the obtained molecules have been previously reported, hence their physical and spectroscopic data are correlated with the literature data [13,14,17].

(**1b**). Yellow solid, mp: 142 °C, yield 65%, FTIR (cm^−1^): 3053 (C_aromatic_–H), 1607 (C=O_keto form_), 1580 (C=O _enol_) 1549 (C=C), 1060 (C_aromatic_–I). HRMS elemental composition C_16_H_12_IO_2_, [M+1]^+^, (error of +0.10 ppm) exact value of 362.98829 Daltons and precise value of 362.98820 Daltons. ^1^H-NMR (400 MHz/CDCl_3_): d 7.92 (1H, H-3), 7.89–7.86 (d, 2H, H-16, H-12), 7.70–7.67 (d, 2H, H-13, H-15), 7.64–7.54 (m, 2H, H-5, H-6), 7.49–7.44 (t, 1H, H-4), 3.92 (s, 2H, H-8). ^13^C-NMR (100 MHz/CDCl_3_): 196.0 C-1, 169.5 C-10, 148.4 C-7, 137.9 C-13; C-15, 137.8 C-2, 134.3 C-9, 133.6 C-5, 129.6 C-12; C-16, 127.6 C-4, 126.1 C-11, 124.6 C-6, 123.7 C-3, 98.3 C-14, 31.1 C-8.

### 2.2. Synthesis and Characterization of Ruthenium Complexes **2a**–**e**

**Scheme 2:**
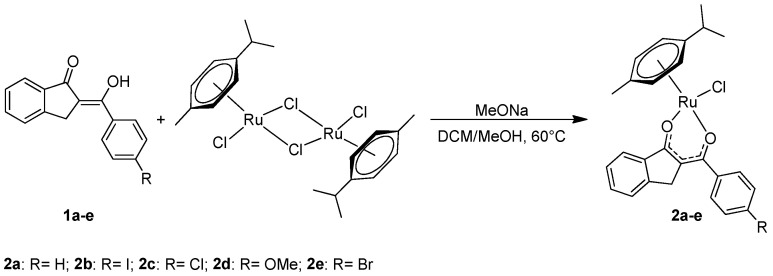
Ruthenium complex’s general reaction, **2a**–**e**.

**Figure 3:**
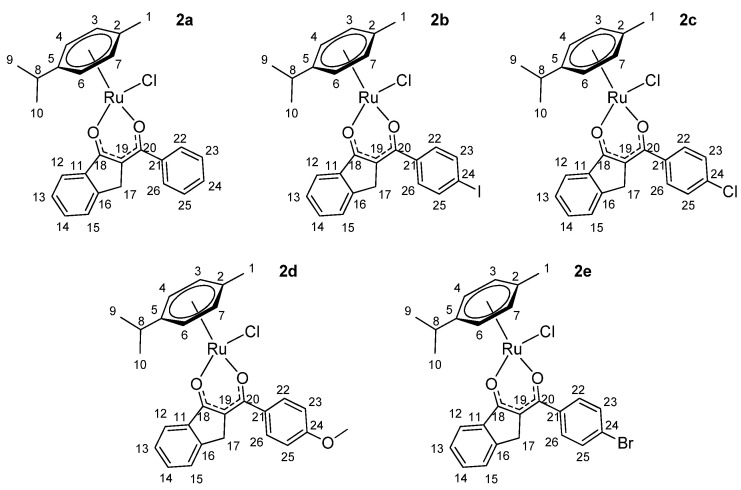
Structure of **2a**–**e**.

The synthesis of the complexes (Scheme 2) was carried out based on a previously reported procedure [17]. [Cy-Ru] (40.0 mg, 0.065 mmol), 2.4 eq of indanone (**1a**–**e**), and 2.2 eq of MeONa were added into a reaction flask; these reagents were dissolved in 20 mL of CH_2_Cl_2_: MeOH (10:1). The reaction mixture was refluxed for 4 h. The solvent was subsequently removed and drops of CH_2_Cl_2_ were added to eliminate insoluble by-products and impurities. The dissolved product was then passed through a celite column. Then the solvent was evaporated, and finally the target product was purified by a silica gel chromatographic column with ethyl acetate/*n*-hexane in a polarity gradient. The structures of all complexes are shown in Figure 3.

(**2a**). Red solid, mp: 184 °C, yield 43%. FTIR (cm^−1^): 3065 (C_aromatic_–H), 1589 (C=O _keto form_), 1555 (C=C), 458 (Ru–O). HRMS elemental composition: C_26_H_25_ClO_2_Ru, [M+1]^+^. ^1^H-NMR (400 MHz/CDCl_3_): 7.87 (d, 1H, *J* = 8 Hz, H-12), 7.68–7.65 (dd, 2H, H-22, H-26), 7.45–7.28 (m, H-23, H-24, H-25), 5.60 (t, *J* =8 Hz, 2H, H-3, H-7), 5.31 (m, 2H, H-4, H-6), 3.72–3.50 (q, 2H, *J* = 28 Hz H-17), 3.05–2.95 (sept, *J* = 12 Hz, 1H, H-8), 2.34 (s, 3H, H-1), 1.47 (s, 3H, H-9), 1.40 (s, 3H, H-10). ^13^C-NMR (100 MHz/CDCl_3_): 183.4 C-18, 180.6 C-20, 146.9 C-16, 140.2 C-11, 140.1 C-21, 131.2 C-24, 129.7 C-14, 128.0 C-23, 127.9 C-25, 126.7 C-13, 124.7 C-15, 123.1 C-12, 108.6 C-5, 99.6 C-19, 97.7 C-2, 83.2 C-3, 83.0 C-7, 79.1 C-4, C-6, 34.8 C-17, 31.0 C-8, 22.5 C-9; C-10, 18.1 C-1.

(**2b**). Red solid, mp: 230 °C, yield 69%. FTIR (cm^−1^): 3049 (C aromatic-H), 1591 (C=O, _keto form_), 1575 (C=O, _enol_), 1548 (C=C), 1056 (C_aromatic_–I), 435 (Ru–O). HRMS elemental composition: C_26_H_24_IO_2_Ru, [M-Cl]^+^, (error of +2.8 ppm) exact value 596.9864 Daltons and precise value of 596.9877 Daltons. ^1^H-NMR (400 MHz/CDCl_3_): 7.90–7.87 (d, 1H, *J* = 8 Hz, H-12), 7.78–7.75 (d, 2H, H-22, H-26), 7.46–7.32 (m, 5H, H-23, H-25, H-13, H-14, H-15), 5.63–5.59 (t, *J* = 8 Hz, 2H, H-3, H-7), 5.34–5.32 (dd, 2H, H-4, H-6), 3.69–3.50 (dd, *J* = 12 Hz, 2H, H-17), 3.03–2.96 (sept, *J* = 8 Hz, H-8), 2.35 (s, 3H, H-1), 1.42–1.39 (d, 6H, H-9, H-10). ^13^C-NMR (100 MHz/CDCl_3_): 179.0 C-18, 148.4 C-16, 137.9 C-11, 137.1 C-23; C-25, 134.3 C-21, 131.4 C-14, 129.6 C-22; C-26, 127.6 C-13, 125.6 C-15, 123.1 C-12, 109.6 C-21, 98.3 C-21, 97.6 C-19, 97.4 C-2, 83.1 C-3, 82.9 C-7, 79.1 C-4, 79.0 C-6, 34.6 C-17, 30.9 C-8, 29.7 C-10, 22.4 C-9, 18.0 C-1.

(**2c**). Red solid, mp: 204 °C, yield 63%. FTIR (cm^−1^): 3042 (C aromatic-H), 1592 (C=O _keto form_), 1578 (C=O, _enol_), 1560 (C=C), 1087 (C_aromatic_–Cl), 452 (Ru–O). HRMS elemental composition: C_26_H_24_Cl_2_O_2_Ru, [M]^+^, (error of +0.8 ppm) exact value 540.0197 Daltons and precise value of 540.0201 Daltons. ^1^H-NMR (400 MHz/CDCl_3_): 7.80 (d, 1H, *J* = 8 Hz, H-12), 7.54–7.52 (d, 2H, H-22, H-26), 7.35 (m, 1H, H-14), 7.35 (d, 1H, H-15), 7.30–7.28 (d, 2H, H-23, H-25), 7.24–7.19 (m, 1H, H-13), 5.53–5.49 (t, *J* = 8 Hz, 2H, H-3, H-7), 5.24–5.21 (m, 2H, H-4, H-6), 3.59–3.41 (dd, *J* = 20 Hz, 2H, H-17), 2.94–2.87 (sept, *J* = 8 Hz, 1H, H-8), 2.24 (s, 3H, H-1), 1.31 (s, 3H, H-9) 1.29 (s, 3H, H-10). ^13^C-NMR (100 MHz/CDCl_3_): 183.8 C-18, 178.9 C-20, 146.7 C-16, 139.8 C-11, 138.5 C-21, 131.4 C-24, 129.3 C-22; C-26, 128.2 C-23; C-25, 126.7 C-13, 124.7 C-15, 123.1 C-12, 108.4 C-5, 99.6 C-2, 97.6 C-19, 83.1 C-3, 82.9 C-7, 79.1 C-4, 79.0 C-6, 34.6 C-17, 30.9 C-8, 22.4 C-9; C-10, 18.0 C-1.

(**2d**). Red solid, mp: 210 °C, yield 70%. FTIR (cm^−1^): 3039 (C _aromatic_–H), 1592 (C=O _keto form_), 1578 (C=O _enol_), 1558 (C=C), 1246 (C_aromatic_–OCH_3_), 1023 (C_aromatic_–OCH_3_), 453 (Ru–O). HRMS elemental composition: C_27_H_27_ClO_3_Ru, [M]^+^, (error of -3.8 ppm) exact value 536.0692 Daltons and precise value of 536.0672 Daltons. ^1^H-NMR (400 MHz/CDCl_3_): 7.80 (d, 1H, *J* = 8 Hz, H-12), 7.64–7.62 (d, 2H, H-22, H-26), 7.34–7.32 (m, 1H, H-15), 7.28–7.24 (m, 2H, H-13, H-14), 6.85–6.83 (dd, 2H, H-23, H-25) 5.53–5.50 (dd, *J* = 4 Hz, 2H, H-3, H-7), 5.25–5.22 (m, 2H, H-4, H-6), 3.79 (s, 3H, OCH_3_), 3.68–3.50 (q, 2H, *J* = 20 Hz H-17), 2.97–2.90 (sept, *J* = 12 Hz, 1H, H-8), 2.27 (s, 3H, H-1), 1.32 (s, 3H, H-9), 1.31 (s, 3H, H-10). ^13^C-NMR (100 MHz/CDCl3): 182.8 C-18, 179.7 C-20, 160.8 C-24, 146.6 C-16, 140.1 C-11, 130.9 C-14, 129.9 C-22; C-26, 126.6 C-13, 124.5 C-15, 122.9 C-12, 113.2 C-23; C-25, 108.2 C-5, 99.5 C-2, 97.3 C-19, 83.2 C-3, 82.9 C-7, 79.1 C-4, 79.0 C-6, 55.4 OCH3, 35.0 C-17, 30.9 C-8, 22.5 C-9; C-10, 18.0 C-1.

(**2e**). Red solid, mp: 202 °C, yield 59%. FTIR (cm-1): 3059 (C_aromatic_–H), 1592 (C=O_keto form_), 1576 (C=O, enol), 1558 (C=C), 1067 (C_aromatic_–Br), 457 (Ru-O). HRMS elemental composition: C_26_H_24_ClBrO_2_Ru, [M]^+^, (error of −0.1 ppm) exact value 583.9692 Daltons and precise value of 583.9691 Daltons. ^1^H-NMR (400 MHz/CDCl_3_): d 7.90–7.87 (d, 1H, *J* = 8 Hz, H-12), 7.64–7.62 (d, 2H, H-23, H-25), 7.46–7.43 (d, 1H,H-14), 7.41–7.38 (dd, m, 3H, H-22, H-26, H13), 7.35–7.32 (d, 1H, H-15), 5.52–5.48 (t, *J* = 8 Hz, 2H, H-3, H-7), 5.23–5.20 (m, 2H, H-4, H-6), 3.57–3.39 (dd, *J* = 20 Hz, 2H, H-17), 2.92–2.85 (sept, *J* = 8 Hz, 1H, H-8), 2.23 (s, 3H, H-1), 1.30 (s, 3H, H-9) 1.28 (s, 3H, H-10). ^13^C-NMR (100 MHz/CDCl_3_): 183.8 C-20, 178.9 C-18, 146.7 C-16, 139.8 C-11, 138.6 C-21, 135.6 C-14, 131.2 C-22; C-26, 129.3 C-23; C-25, 128.2 C-22, C-26, 126.7 C-13, 124.6 C-15, 123.1 C-12, 108.4 C-5, 99.5 C-2, 97.6 C-19, 83.2 C-3, 82.9 C-7, 79.1 C-4, 79.0 C-6, 34.6 C-17, 30.9 C-8, 22.4 C-9; C-10, 18.0 C-1.

### 2.3. Deposit and Characterization of Hybrid Films

The polymer hybrid films were deposited on glass and on glass substrates coated with FTO (Fluorine-doped Tin Oxide) and n-type silicon wafer single side polish. The glass and glass coated with FTO substrates were previously washed in an ultrasonic bath with chloroform, isopropanol, and acetone. The n-silicon was washed with hydrogen peroxide, isopropanol, and acetone. A ruthenium complex was employed as a semiconductor material, hence it was embedded in PMMA polymer to form a hybrid film. Every polymer hybrid film was produced by drop-casting with a dispersion of 0.6 mL of the poly(methyl methacrylate) (PMMA; [CH_2_C(CH_3_)(CO_2_CH_3_)]n) and ruthenium complex from a dilution of 10 wt% in chloroform. The mixture was dispersed using the G560 shaker (Bohemia, New York, NY, USA). After the drop-casting the polymer hybrid film was brought to 55 °C for 5 min in the drying oven Briteg SC-92898 (Instrumentos Científicos, S.A de C.V. Puebla, México). The hybrid films on the n-silicon substrates were used for infrared spectrophotometric evaluation using a Nicolet iS5-FT spectrophotometer (Thermo Fisher Scientific Inc., Waltham, MA, USA). The roughness, topography, thickness, and some mechanical properties of the polymer hybrid films on silicon substrate were investigated with an atomic force microscope (AFM) using an Ntegra platform (Nanosurf AG, Liesta, Switzerland) in contact mode; subsequently, the images were analyzed with the Gwyddion 2.65 software. To obtain the mechanical parameters of the hybrid films, force spectroscopy was used, which is a method used in Nanosurf atomic force microscopes and refers to a measurement in which the cantilever approaches and indents the planar film surface and then withdraws. The cantilever deflection vs. piezo movement is measured, and this can be converted to a force vs. tip sample separation measurement that provides mechanical information about the hybrid film. The AFM force curves can be used for various mechanical parameters extraction, including the relationship of adhesion force (F), stress (σ), strain (ε), and indentation depth for Knoop hardness (HK). The absorbance and transmittance were obtained for films deposited on glass substrates, using an ultraviolet-visible spectrophotometer 300 Unicam (Thermo Fisher Scientific Inc., Waltham, MA, USA). The electrical properties in the hybrid films were obtained using a QUESA-1200 system with LED light sourced from the “TFSC instrument” Inc. (Intercovamex, S.A. de C.V., Cuernavaca, Morelos, Mexico).

## 3. Results and Discussion

### 3.1. Synthesis and Characterization of Ru Complexes

The ruthenium complexes **2a***–***e** were obtained by the reaction between [Cy-Ru] with the correspondient ligand in CH_2_Cl_2_:MeOH (1:1) as the solvent in a stochiometric ratio. The resulting complexes were obtained in a 43–70% range yield (moderate to good yields) as orange solids; the target molecules were characterized by FTIR, MS, ^1^H-NMR, and ^13^C-NMR, in complement with HSQC and HMBC experiments (suplementary data). Through FTIR spectrosphotometry, the products showed a typical band for a carbonyl vibration, also a band corresponding to a C=C double bond and the characteristic vibrations of Ru–O, in addition to the corresponding C-halogen deformation, were also present. Mass spectrometry allowed obtaining the molecular ion of a new molecule (**1b** and **2a***–***e**) using a FAB+, DART and, ESI as ionization sources, confirming the compound formed, in which the same fractionation pattern was also observed for each one. Two-dimensional experiments for a more unequivocal determination were performed for the NMR of 1H, 13C, and, in some cases, an HSQC and HMBC. Moreover, in general, 1H expected signals and multiplicities for each compound were appropiately observed; related to ^13^C data, the typical shifts were conveniently correlated with those previously observed in other developed complexes by our research group toghether with those corresponding to the cymene fragment.

**Figure 4:**
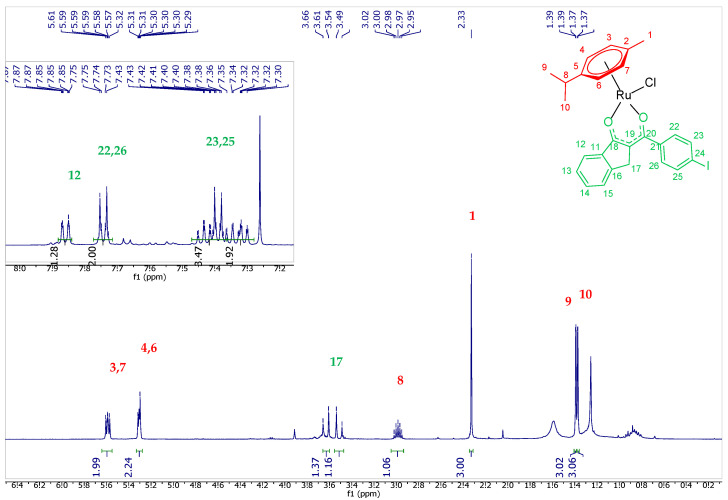
^1^H-NMR spectra of **2b**. Green hydroxymethilidene indanone **1b** fragment, red p-cymene fragment.

As an example, a summary of the characterization for the target **2b** is presented: It was observed that the characteristic ^1^H-NMR signals (Figure 4) for the p-cymene fragment between 5.61–5.57 and 5.32–5.29 ppm were assigned to H-3, H-7, H-6 and H-4, respectively. Additionally, both methyls (H-9, H-10) in p-cymene displayed a shift of 1.39–1.37 ppm, while the CH_2_ (H-17) from the indanone was observed at 3.66–3.49 ppm. Lastly, the signals in the 7.75–7.73 and 7.34–7.30 ppm corresponded to the p-substituted aromatic ring with iodine [17].

**Figure 5:**
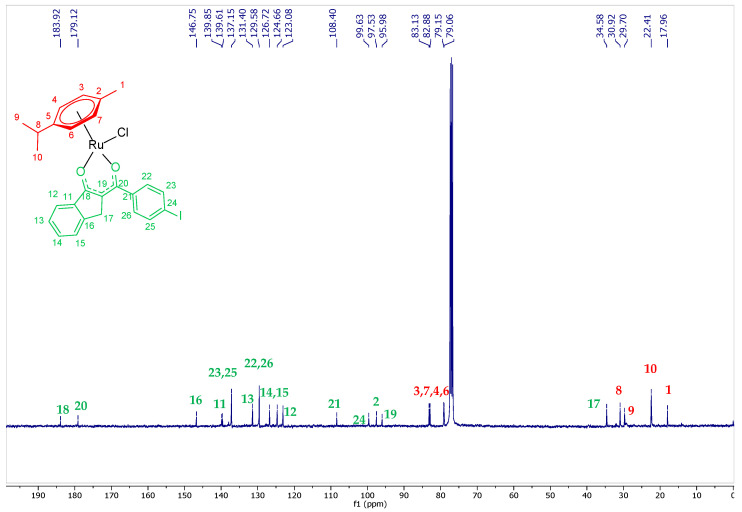
^13^C-NMR spectra of **2b**. Green hydroxymethilidene indanone **1b** fragment, red p-cymene fragment.

Related to the ^13^C-data (Figure 5), characteristic signals for the carbonyl carbons C-18 and C-20 are present at 183.9 and 179.1, respectively. In addition, the quaternary carbons C-16, C-11, C-21, C-24, and C-19 appear at 146.7, 139.8, 108.4, 99.6 and 95.9 ppm, respectively. Finally, the following signals were observed: 137.1 and 129.6 ppm for the p-substituted aromatic system with iodine, together with 83.1 and 79.1 ppm for the quaternary carbons in the p-cymene fragment [17].

In the FTIR (Supplementary Materials), ligand **1b** presents characteristic bands of C=O at 1607 and 1580 cm^−1^, and, in the case of complex **2b**, these bands appeared at 1591 and 1575 cm^−1^, respectively, which indicates a carbonyls range when they are complexed with ruthenium [27]. Finally, in the case of the mass spectrometric data acquired by ESI^+^ (Supplementary Materials), an intense peak was observed at m/z 596, which is characteristic of the loss of an Cl that is coordinated to Ru.

### 3.2. Characterization of Hybrid Films

**Figure 6:**
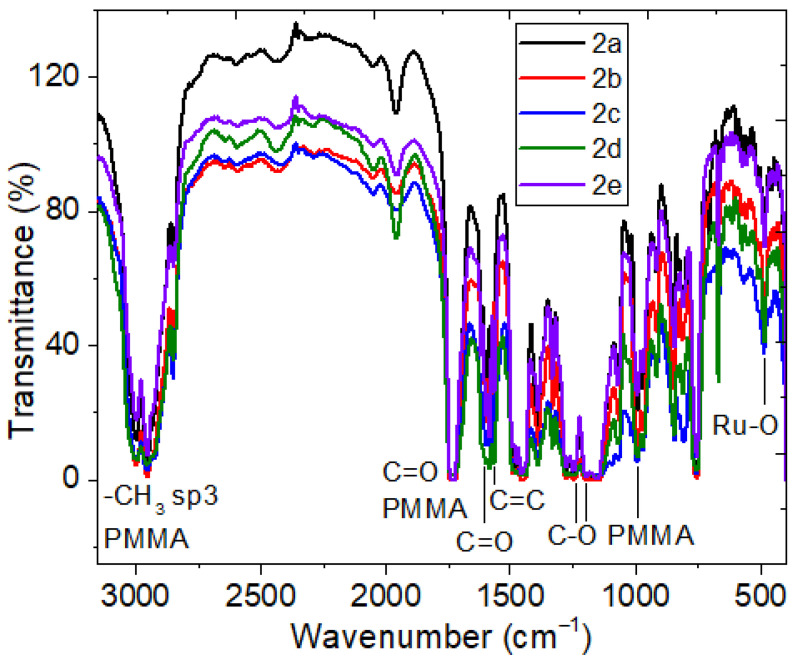
IR spectrum of hybrid films based on ruthenium complexes and PMMA matrix.

IR spectroscopy in hybrid films was carried out in order to verify if the ruthenium complexes suffered chemical decomposition due to the drop-casting process. The file with the SMIR support material includes a comparison of the spectrum of each of the complexes in the KBr pellet and in its hybrid film. In these spectra the signals corresponding to the ruthenium complexes and the small shifts between the values are mainly due to the presence of PMMA in the hybrid films. Additionally, Figure 6 shows the IR spectra for the polymer hybrid films, in which the bands of C=O_keto form_, C=O_enol_, C=C, and Ru–O are evident. However, the band corresponding to C aromatic–H (3049–3065 cm^−1^) was not distinguished, due to the presence of the intense PMMA bands corresponding to (i) the asymmetric stretching vibration for CH in aliphatic group (2850 cm^−1^) and (ii) the combination band involving CH and CH_3_ group (2951 cm^−1^) [28]. Other prominent PMMA bands present in the spectrum correspond to the C–O moiety at 1241 cm^−1^ (symmetric vibration for C–C–O combined with CH deformation), 1193 cm^−1^ (asymmetric vibration for C–O–C group with internal CH deformation) and 987 cm^−1^ (C–O–C combined with OCH_3_ group). Finally, the band at 1727 cm^−1^ was also observed, corresponding to the symmetric stretching vibration for C=O group [28]. Table 1 presents in detail all the bands and their assignments for each hybrid film. The presence of all the bands confirms the incorporation of ruthenium complexes in the polymeric matrix and, in addition, this information also suggests that the ruthenium complexes are structurally unchanged during the preparation of hybrid films.

**Figure 7:**
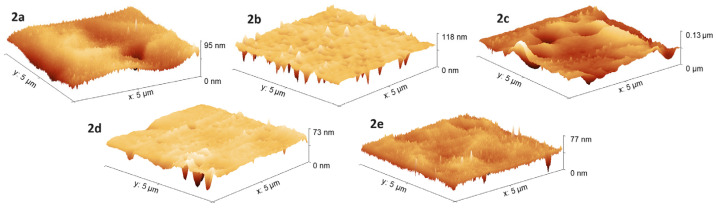
AFM images of hybrid films based on ruthenium complexes **2a**–**2e** and PMMA matrix.

The topography of the films was characterized using AFM in tapping mode and the 3D images are shown in Figure 7. In the 3D images, it was observed that films **2b** and **2d** are the ones that present greater homogeneity, and an irregular topography was observed with the presence of large holes in the films in complexes **2a**, **2c**, and **2e**. On the other hand, Table 2 summarizes the values of RMS (Root Mean Square) and Ra (Roughness Average) roughness. From this table, the low roughness of films **2b**, **2d**, and **2e** is evident, which is favorable if in the future it is intended to use these films in the manufacture of organic electronic devices, in which charge transport must be carried out efficiently. Efficient charge transport in devices requires that these charges be able to move quickly from one molecule to another, and not be trapped or dispersed. This is because organic molecules interact with each other, mainly through van der Waals forces and Coulombic interactions [29]. The low roughness in films **2b**, **2d** and **2e** can be related to better molecular packing along the film surface, and, thanks to this, adequate electronic coupling between the molecules could be generated. On the contrary, the high roughness of the complex **2c** hybrid film is related to a low level of stacking between the molecules that make up its surface. Due to the above, its potential for charge transport decreases, since as mentioned above, it is related to interactions between neighboring molecules [29]. Furthermore, the presence of large holes on the surface of film **2c** favors charge dissipation.

On the other hand, Table 2 shows the stress (σ), the strain (ε), and the Knoop hardness (HK) calculated for force spectroscopy. The σ and ε are similar for all films, while the Knoop hardness (HK) maximum and minimum values change significantly in the hybrid film **2a** and in hybrid film **2e**, respectively. If it is considered that all the polymer hybrid films were manufactured with the same conditions and stoichiometric ratios, then it can be concluded that the changes between them are due to the type of ruthenium complex used and its ability to integrate with the PMMA polymer matrix. The mechanical parameters obtained by AFM are an indication that despite the heterogeneity of the hybrid films, their good properties can favor their mechanical resistance under service conditions when used as semiconductor materials.

**Figure 8:**
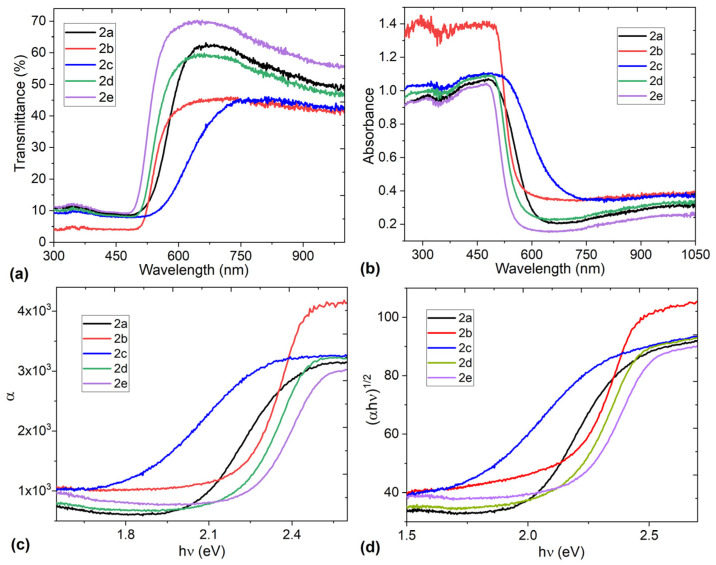
(**a**) Transmittance, (**b**) absorbance, (**c**) relationship between the absorption coefficient, α, the photon energy, hν, and (**d**) Tauc curves for the films in the ruthenium complexes.

The films in the ruthenium complexes embedded in the PMMA were optically characterized and the main optical parameters are shown in Figure 8. With respect to the transmittance shown in Figure 8a, it was observed that the maximum transparency was obtained at wavelengths greater than 590 nm. The film with complex **2e**, which had the bromide radical, had the highest transparency of 70%, followed by film **2a** at 63%, then the film with complex **2d** at 59%, and finally, the films with complexes **2b** and **2c**, which had a similar transparency at around 45%. The greatest transparency for the films was obtained in a very short range of wavelengths and from 770 nm all of the films had a significant decrease in transmittance. It is important to mention that the **2e** film, with its high transmittance, could be used as a transparent anode in organic solar cells. In this type of device, an anode semi-transparent to radiation in the visible spectral range is required, and hybrid polymer matrix films with conductive materials such as graphene or carbon nanotubes have been used [30,31,32].

With respect to the absorbance shown in Figure 8b, a zone of great absorption is observed at low wavelengths. In addition, the UV–Vis spectra show small differences due to the substituents in the ruthenium complexes; the radicals gives rise to a slight displacement of the spectral bands as a result of the different electronic effects exerted by these substituents. However, the spectra of all the films show a similar aspect, which can be attributed to transitions of the same nature. This type of behavior has occurred in other indanone derivatives such as iron(III) complexes of 2-benzylidene-1-indanone derivatives [13], and the organotin(IV) complexes derived from aryl hydroxymethylidene indanones [14]. The ruthenium complexes have absorption bands assigned to the following electronic transitions: d–d transitions between d orbitals of ruthenium, intraligand transitions π–π* arising from molecular orbitals localized in the ligand and charge transfer transitions involving transitions from the ligand to the metal ion or from the metal ion to the ligand [26,27]. The band appearing around 264 nm seems to correspond to a π–π* transition in the enolate ring and the one around 312 nm comes from π–π and π–π* intraligand transitions. The band around 407–487 nm is due to charger transfer from the ligand to the metal ion [26,27]. The π interaction in the enolate ring is influenced by the type of substituent, as manifested in the observed spectral differences [25]. The greatest absorption was that of the film with complex **2b**, which has the iodide radical in its structure. This film could be used as an active layer in organic solar cells, which require that their active layers be responsible for the absorption of radiation, and where the charge carriers that give rise to the photocurrent are generated. This layer is normally made up of semiconducting materials.

A parameter that provides very useful information to quantify the semiconductor properties of this type of complexes is the energy difference between their HOMO (highest occupied molecular orbital) and LUMO (lowest unoccupied molecular orbital) frontier orbitals. This is because charge transport in the semiconductor involves the movement of electrons along the LUMO and the transport of holes within the HOMO. Using UV–Vis spectrophotometry, the value of the optical HOMO–LUMO gap (E_opt_) is estimated. This optical band gap is attributed to the lower energy transition that takes place by absorption of a photon. Normally, organic semiconductors have E_opt_ lower than 3 eV, the value depends on the type of molecule and its structure [33,34], and there are also low band gap organic semiconductors with values lower than 1.5 eV [35]. To calculate the E_opt_, different methods can be applied, one of them consists of the extrapolation of the linear section of the lower energy skirt of the absorption coefficient (α), up to the cut-off point with the photon energy axis (hν). The E_opt_ values obtained in Figure 8c, are summarized in Table 3 and follow the sequence: **2c** < **2a** < **2d** < **2b** < **2e**. It is important to note that all values are in the range of organic semiconductors [33,34], so all hybrid films present semiconducting behavior. These results are interesting, because the optical band gaps obtained are low, although the hybrid films present structural disorder due to the weakness of their non-covalent interactions, and due to the environment of each ruthenium complex molecule, which is not identical to that of other molecules in the film.

On the other hand, the optical band gap values can also be interpreted using the Tauc model, according the relation [36,37] written as:

(1) $$\;\;\alpha =\alpha_{0}\;{\left(hv-E_{Tauc}\right)}^{r}$$

where E_Tauc_ is the Tauc band gap and r determines the type of transitions (for example, r = 2 for indirect transitions in amorphous films like the polymer hybrid films). The band gap is determined from the Tauc extrapolation [36,37], the intercept on the energy axis of the linear fit of the larger energy data, in a plot of (αhν)^1/2^ vs. hν in Figure 8d. The Tauc band gap for the hybrid films was found to be between 1.68 and 2.17 eV (see Table 3). These values are slightly lower than E_opt_, and they are significantly lower than those obtained for other complexes derived from indanones of iron (2–2.1 eV) [13], tin (2.86–2.95 eV) [14], vanadium (2.6–2.7 eV) [14], copper (2.76–2.97 eV) [16], and uranium (2.4–2.93 eV) [17]. The band gap obtained gives an indication of the adequate semiconductor behavior that all hybrid films have. It is also important to mention that the substituent in the ruthenium complex is responsible for the change in the band gap values. Although the band gap is very similar in all hybrid films, the lowest value was obtained by the film in complex **2c**, which has the chloride substituent in its structure. This is probably due to the small size and high electronegativity of the chloride, which is reflected in its high ability to attract electrons, with respect to the rest of the ruthenium complexes.

Finally, it is important to consider that in the hybrid films there are a number of traps and defects that can be indirectly evaluated through the Urbach energy (E_U_). The E_U_ corresponds to the width of the band tail, which is related to localized states within the energy gap, caused by structural defects [38]. The E_U_ can be determined according to the expression [39]:

(2) $$\alpha =A_{a}exp\left(\frac{hv}{E_{U}}\right)$$

**Figure 9:**
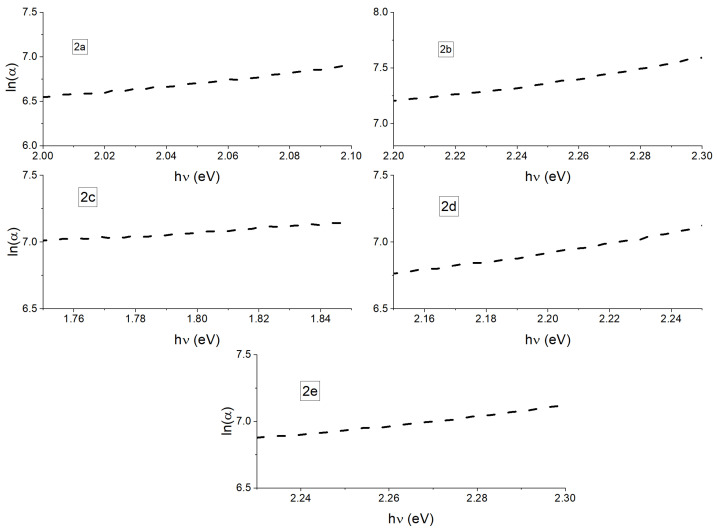
Relation between ln(α) vs. hν energy for polymeric hybrid films with **2a**–**2e** ruthenium complexes.

In addition to the parameters defined above, A_a_ is a constant of the material that conforms to α at the energy gap. The values of the E_U_ were determined from the reciprocal of the slope from this linear relation and have been recorded in Figure 9. The value of E_U_ is zero in a perfect semiconductor [39] and in this study the values are less than 0.5 eV, which is an indication that although the films have several phases due to the mixture between the PMMA and the ruthenium complexes, the interaction between the two materials allows the transport of charges through their interfaces. Furthermore, the band gap values obtained, as well as the transmittance and absorbance presented by the hybrid films, are indicative of the adequate semiconductor behavior that they could have as transparent electrodes or active layers, in devices such as, for example, organic solar cells [2], flexible displays [40,41], or polymer light-emmitting diodes [42].

Finally, in order to evaluate the current density (J) transported at room temperature in the hybrid films, glass/FTO/hybrid film/Ag devices were manufactured using FTO as an anode and Ag as the cathode. The results are summarized in Table 4, and it was observed that the device with the hybrid film of compound **2c** is the one that generates the greatest charge transport. The above is to be expected because this film is the one with the smallest band gap. The electrical conductivity (σ) was also determined, and the results are found in Table 4; these σ values are within the range of organic semiconductors (10^−6^ to 10^2^ S/cm) [43]. However, in the future, it is necessary to carry out more complete studies on the electrical behavior of these films, which will allow us to determine the type of specific applications in organic optoelectronic devices that they may have. Furthermore, to mention some of the aspects that could be carried out in future studies, the electrical conductivity in devices with these hybrid films can be increased with the inclusion of electron transport layers, hole transport layers, or by modifying the thicknesses.

## 4. Conclusions

A new indanone (**1b**) was obtained, which was used together with other previously reported indanones to obtain, with 43–70% yields, four new piano-stool ruthenium complexes (**2a**–**e**) characterized by conventional spectroscopic methods; it is important to highlight, that to our knowledge, there are almost no studies reported on their potential application as semiconductors and subsequently integrated into a PMMA matrix to form hybrid polymer films. The obtained hybrid films disclose a band gap, both optical (1.73–2.24 eV) and Tauc (1.68–2.17 eV), which places them within the range of organic semiconductors being excellent candidates to be used in organic electronics. The device with the hybrid film of compound **2c** is the one that presented the lowest band gap and the highest transported current density, which makes it the best candidate for applications in optoelectronic devices.

## Figures and Tables

**Table 1:** Assignments of ruthenium complexes and polymer hybrid films IR spectra.

Hybrid Film	(C=O_keto form_) cm^−1^	(C=O_enol_) cm^−1^	(C=C) cm^−1^	(Ru–O) cm^−1^	PMMA: (C–H) cm^−1^	PMMA: (C–O) cm^−1^	PMMA: (C=O) cm^−1^
**2a** (film)	1594		1568	457	2950, 2847	1242, 1193, 989	1732
**2a** (KBr)	1589		1555	458			
**2b** (film)	1595	1577	1553	432	2952, 2847	1243, 1190, 989	1724
**2b** (KBr)	1591	1575	1548	435			
**2c** (film)	1590	1581	1562	457	2950, 2849	1243, 1190, 990	1733
**2c** (KBr)	1592	1578	1560	452			
**2d** (film)	1590	1580	1561	450	2953, 2848	1243, 1190, 989	1721
**2d** (KBr)	1592	1578	1558	453			
**2e** (film)	1595	1579	1560	450	2950, 2848	1243, 1190, 990	1732
**2e** (KBr)	1592	1576	1558	457			

**Table 2:** Roughness, mechanical parameters, and thickness of films with ruthenium complexes.

Film	RMS Roughness (nm)	Ra Roughness (nm)	Maximum Stress, σ (MPa)	Strain, ε	Knoop Hardness, HK	Thickness (μm)
**2a**	10.42	8.14	10.5	0.96	18.42	5.8
**2b**	8.33	5.03	10.3	0.91	4.92	6.1
**2c**	15.91	11.78	10.1	0.91	4.52	5.2
**2d**	5.48	3.07	10.1	0.91	4.52	7.5
**2e**	4.41	3.40	10.3	0.87	2.14	5.3

**Table 3:** Optical band gap, Tauc band gap, and Urbach energy for the hybrid films.

Hybrid Film	E_opt_ (eV)	E_Tauc_ (eV)	E_U_ (eV)
**2a**	2.05	2.01	0.29
**2b**	2.21	2.15	0.27
**2c**	1.73	1.68	0.50
**2d**	2.20	2.14	0.30
**2e**	2.24	2.17	0.36

**Table 4:** Current density (J) and electrical conductivity (σ) for glass/FTO/hybrid film/Ag devices.

Sample	J (mA/cm^2^)	σ (S/cm)
Device **2a**	0.031335237	4.89 × 10^−6^
Device **2b**	0.031123662	4.85 × 10^−6^
Device **2c**	0.069826411	1.09 × 10^−5^
Device **2d**	0.031383043	4.9 × 10^−6^
Device **2e**	0.031766458	5.0 × 10^−6^

## Data Availability

The data are contained within the article and Supplementary Materials.

## Supplementary Materials

The following supporting information can be downloaded at: https://www.mdpi.com/article/10.3390/polym16101338/s1, Figure S1: FTIR spectrum of ligand **1b**, Figure S2: MS-DART+ spectrum of ligand **1b**, Figure S3: elemental analysis of ligand **1b**, Figure S4: 1H-NMR spectrum of ligand **1b** in CDCl3 400 MHz, Figure S5: 13C-NMR spectrum of **1b** in CDCl3 400 MHz, Figure S6: FTIR spectrum of complex **2a**, Figure S7: MS-FAB+ spectrum of complex **2a**, Figure S8: Elemental analysis of complex **2a**, Figure S9: 1H-NMR spectrum of **2a** in CDCl3 400 MHz, Figure S10: 13C-NMR spectrum of **2a** in CDCl3 400 MHz, Figure S11: FTIR spectrum of complex **2b**, Figure S12: MS-ESI spectrum of complex **2b**, Figure S13: Exact mass by MS-ESI of complex **2b**, Figure S14: 1H-NMR spectrum of complex **2b** in CDCl3 400 MHz, Figure S15: 13C-NMR spectrum of complex **2b** in CDCl3 400 MHz, Figure S16: IR spectrum of complex **2c**, Figure S17: MS-FAB+ spectrum of complex **2c**, Figure S18: Elemental analysis of complex **2c**, Figure S19:1H-NMR spectrum of **2c** in CDCl3 400 MHz Figure S20: 13C-NMR spectrum of **2c** in CDCl3 400 MHz, Figure S21: HSQC 2D spectrum of complex **2c**, Figure S22: FTIR spectrum of complex **2d**, Figure S23: MS-FAB+ spectrum of complex **2d**, Figure S24: Elemental analysis of complex **2d**, Figure S25: 1H-NMR spectrum of complex **2d** in CDCl3 400 MHz, Figure S26: 13C-NMR spectrum of **2d** in CDCl3 400 MHz, Figure S27: HSQC 2D spectrum of complex **2d** in CDCl3, Figure S28: FTIR spectrum of complex **2e**, Figure S29: MS-FAB+ spectrum of complex **2d**, Figure S30: Elemental analysis of complex **2e**. Figure S31: 1H-NMR spectrum of complex **2e** in CDCl3 400 MHz, Figure S32: 13C-NMR spectrum of **2e** in CDCl3 400 MHz. Figure IR1: FTIR spectrum of complex **2a** (KBr), Figure IR2: FTIR spectrum of complex **2a** (hybrid film), Figure IR3: FTIR spectrum of complex **2b** (KBr), Figure IR4: FTIR spectrum of complex **2b** (hybrid film), Figure IR5: IR spectrum of complex **2c** (KBr), Figure IR6: FTIR spectrum of complex **2c** (hybrid film), Figure IR7: FTIR spectrum of complex **2d** (KBr), Figure IR8: FTIR spectrum of complex **2d** (hybrid film), Figure IR9: FTIR spectrum of complex **2e** (KBr), Figure IR10: FTIR spectrum of complex **2e** (hybrid film).
