# Supplementary material for: Synthesis of New Ruthenium Complexes and Their Exploratory Study as Polymer Hybrid Composites in Organic Electronics
Source: Polymers (Basel). 2024 May 9;16(10):1338. doi: 10.3390/polym16101338 (PMC11125087; doi:10.3390/polym16101338)

Synthesis of new ruthenium complexes and their exploratory study as organic semiconductors

Ricardo Ballinas-Indilí [1], María Elena Sánchez-Vergara [2,*], Saulo C. Rosales-Amezcua [3], Joaquín André Hernández Méndez [2], Byron Lópéz-Mayorga [4], René Miranda Ruvalcaba [1], and Cecilio Álvarez-Toledano [3]

[1] Departamento de Ciencias Químicas, Facultad de Estudios Superiores Cuautitlán Campo 1, Universidad Nacional Autónoma de México, Avenida 1o de Mayo s/n, Colonia Santa María las Torres, Cuautitlán Izcalli 54740, Mexico; mirruv@yahoo.com.mx (R.M.-R.)

[2] Facultad de Ingeniería, Universidad Anáhuac México, Av. Universidad Anáhuac 46, Col. Lomas Anáhuac, Huixquilucan 52786, Mexico

[3] Instituto de Química, Universidad Nacional Autónoma de México, Circuito Exterior s/n, Ciudad Universitaria, Mexico City 04510, Mexico; cecilio@unam.mx (C.Á.-T.)

[4] Escuela de Química, Facultad de Ciencias Químicas y Farmacia, Universidad de San Carlos de Guatemala, 11 avenida, Ciudad de Guatemala 01012, Guatemala, byron.lopez@profesor.usac.edu.gt (B.L.-M)

* Correspondence: elena.sanchez@anahuac.mx

Content

Ligand **1b** (FTIR, MS, Elemental analysis ^{1}HNMR, ^{13}CNMR) …………………………… S1-S5

Complex **2a** (FTIR, MS, Elemental analysis, ^{1}HNMR, ^{13}CNMR) ………………………… S6-S10

Complex **2b** (FTIR, MS, Elemental analysis, ^{1}HNMR, ^{13}CNMR) ………………………… S11-S15

Complex **2c** (FTIR, MS, Elemental analysis, ^{1}HNMR, ^{13}CNMR, HSQC) ………………… S16-S21

Complex **2d** (FTIR, MS, Elemental analysis, ^{1}HNMR, ^{13}CNMR, HSQC) ………………… S22-S26

Complex **2e** (FTIR, MS, Elemental analysis, ^{1}HNMR, ^{13}CNMR) ………………………….. S27-S32

Figure S1: FTIR spectrum of ligand **1b**.

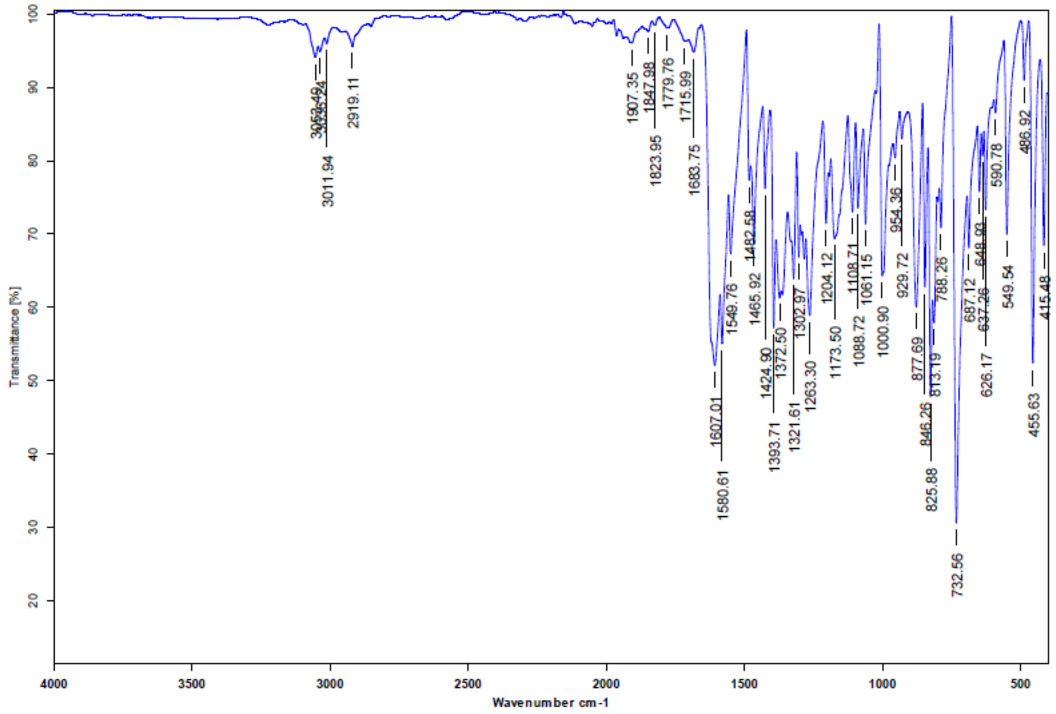

Figure S2: MS spectrum of ligand **1b**.

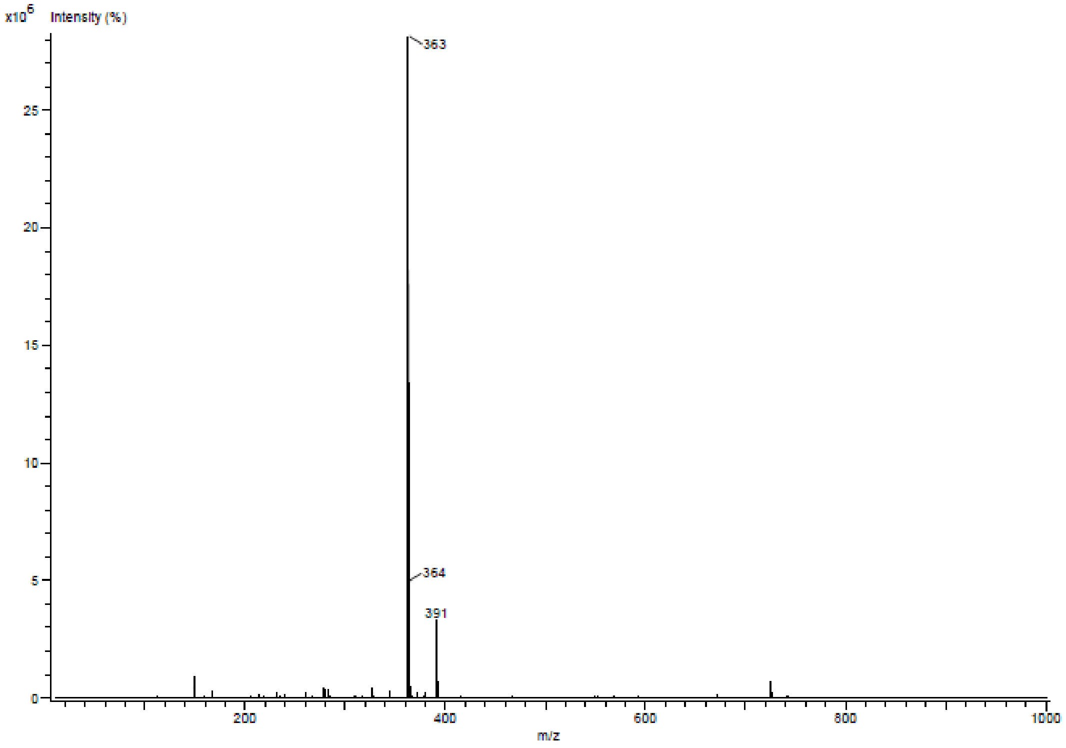

Figure S3: HRMS of ligand **1b**.

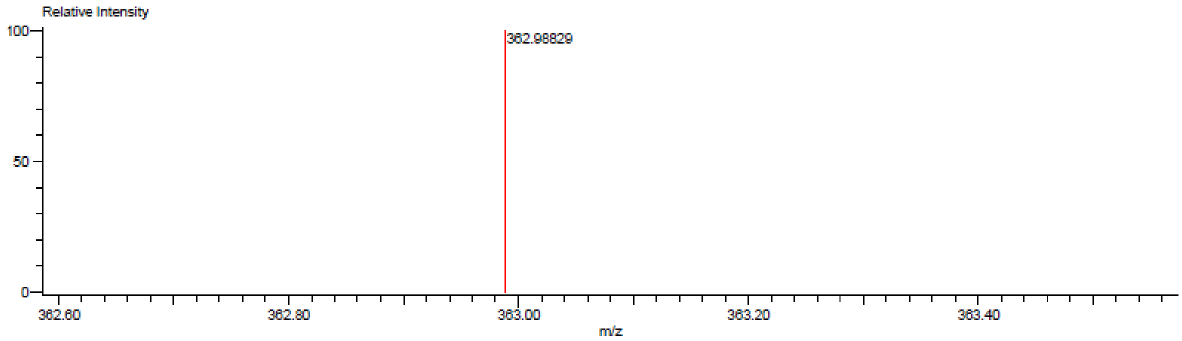

Mass	Intensity	Calc. Mass	Mass Difference (mmu)	Mass Difference (ppm)	Possible Formula	Unsaturation Number
362.98829	4215.56	362.98820	0.10	0.27	$^{12}C_{16}{}^{1}H_{12}{}^{127}I_{1}{}^{16}O_{2}$	10.5

Figure S4: ^{1}H-NMR spectrum of ligand **1b** in $CDCl_3$ 400 MHz.

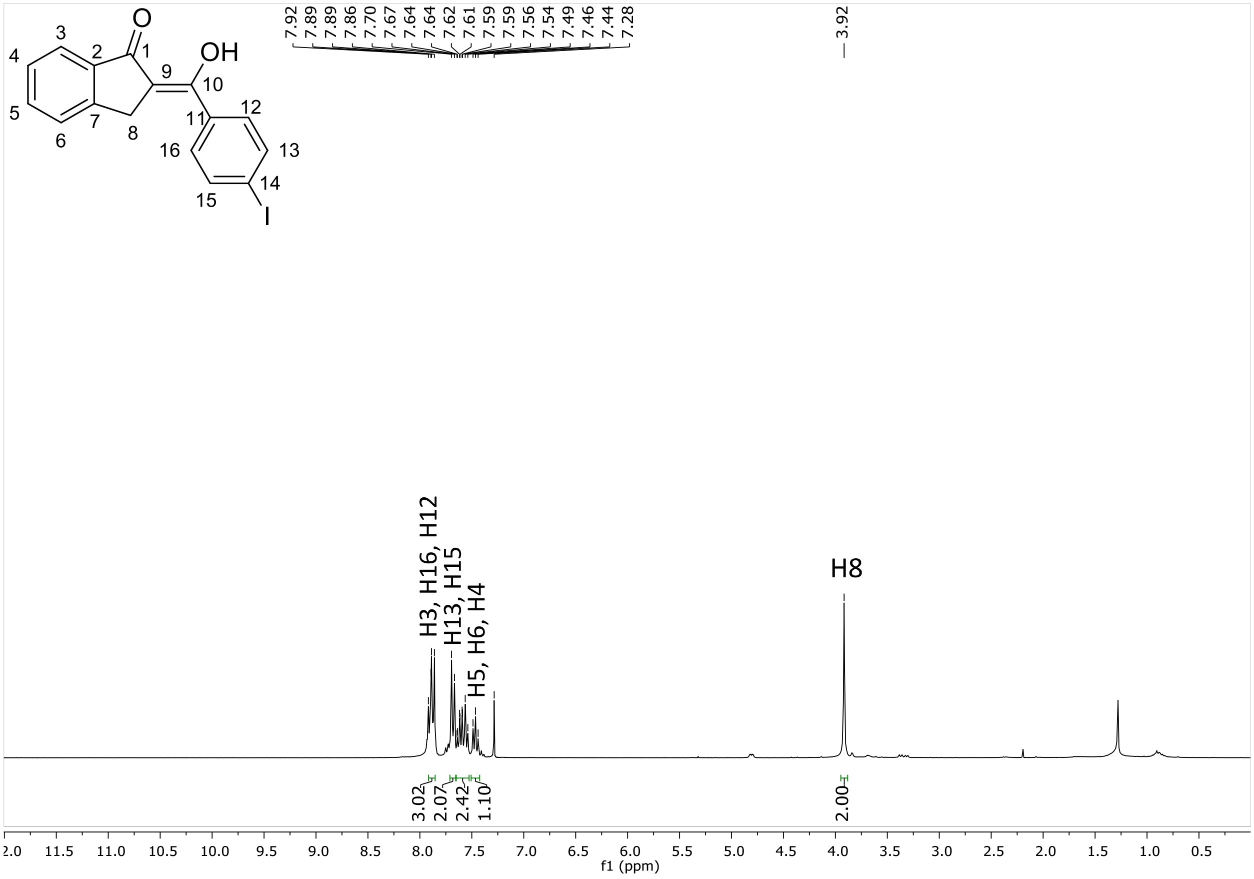

Figure S5: ^{13}C-NMR spectrum of **1b** in $CDCl_3$ 400 MHz:

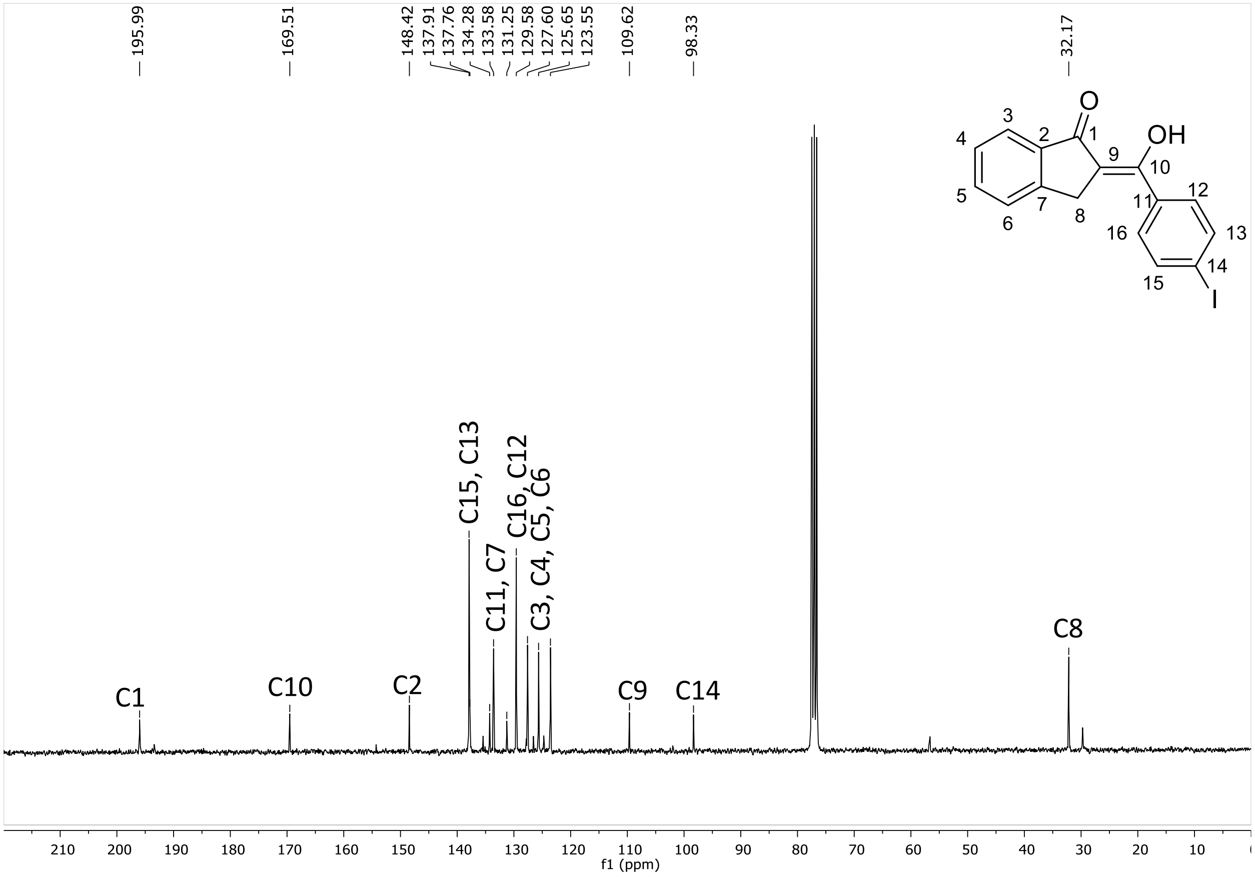

Figure S6: FTIR spectrum of complex **2a**.

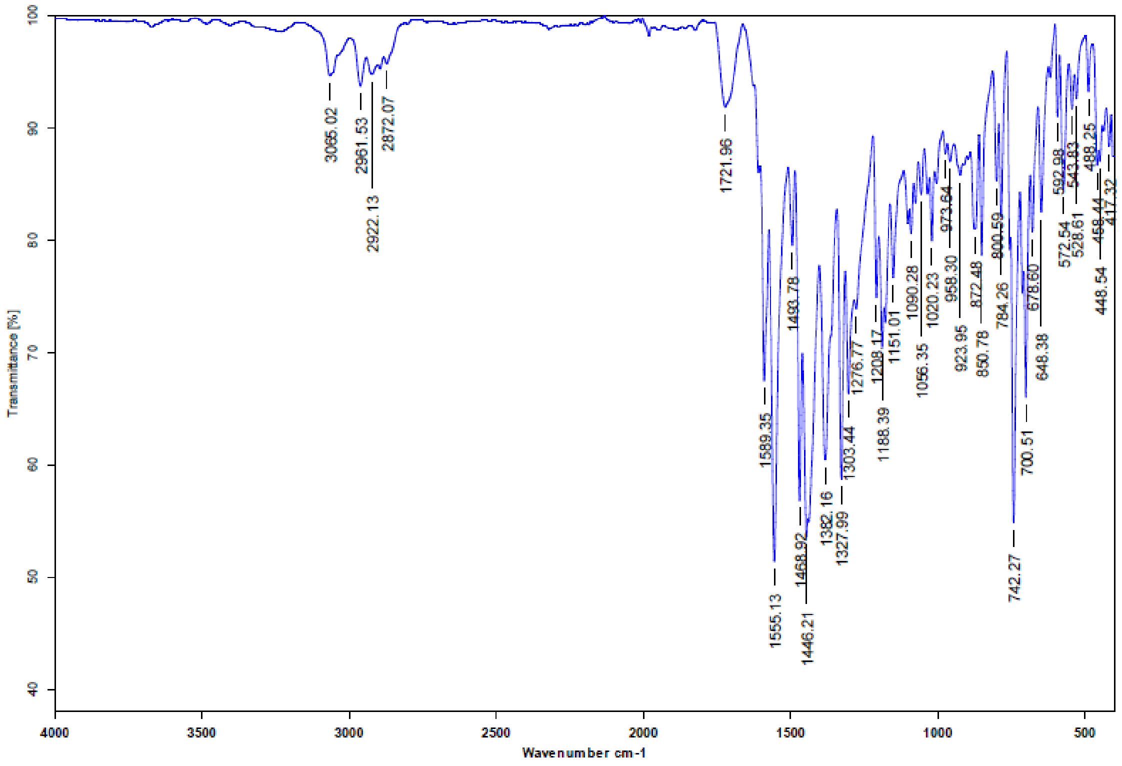

Figure S7: MS spectrum of complex **2a**.

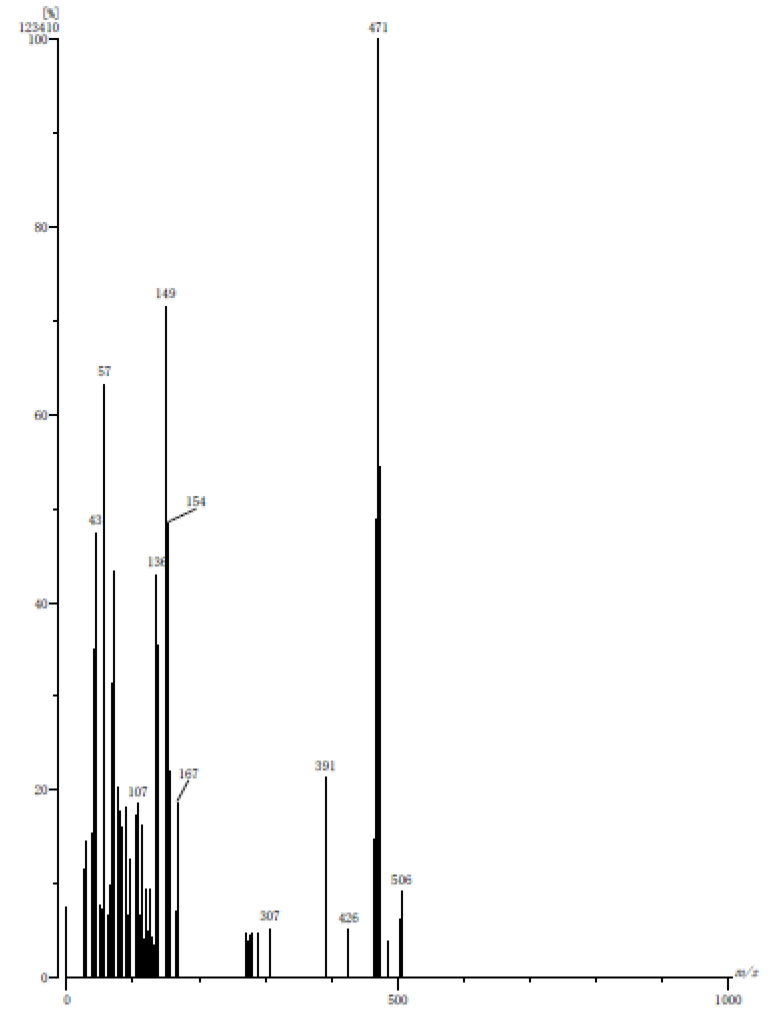

Figure S8: HRMS of complex **2a**.

Inlet : Direct Ion Mode : FAB+
RT : 6.71 min Scan# : (141,142)
Elements : C 26/0, H 29/0, Cl 1/0, O 2/0, Ru 1/0
Mass Tolerance : 1000ppm, 10mmu if m/z > 10
Unsaturation (U.S.) : 0.0 - 20.0

	Observed m/z	Int%							
	506.0600	100.00							
	Estimated m/z	Err[ppm / mmu]	U.S.	C	H	Cl	O	Ru
1	506.0587	+2.7 / +1.3	14.5	26	25	1	2	1

Figure S9: ^{1}H-NMR spectrum of **2a** in $CDCl_3$ 400 MHz:

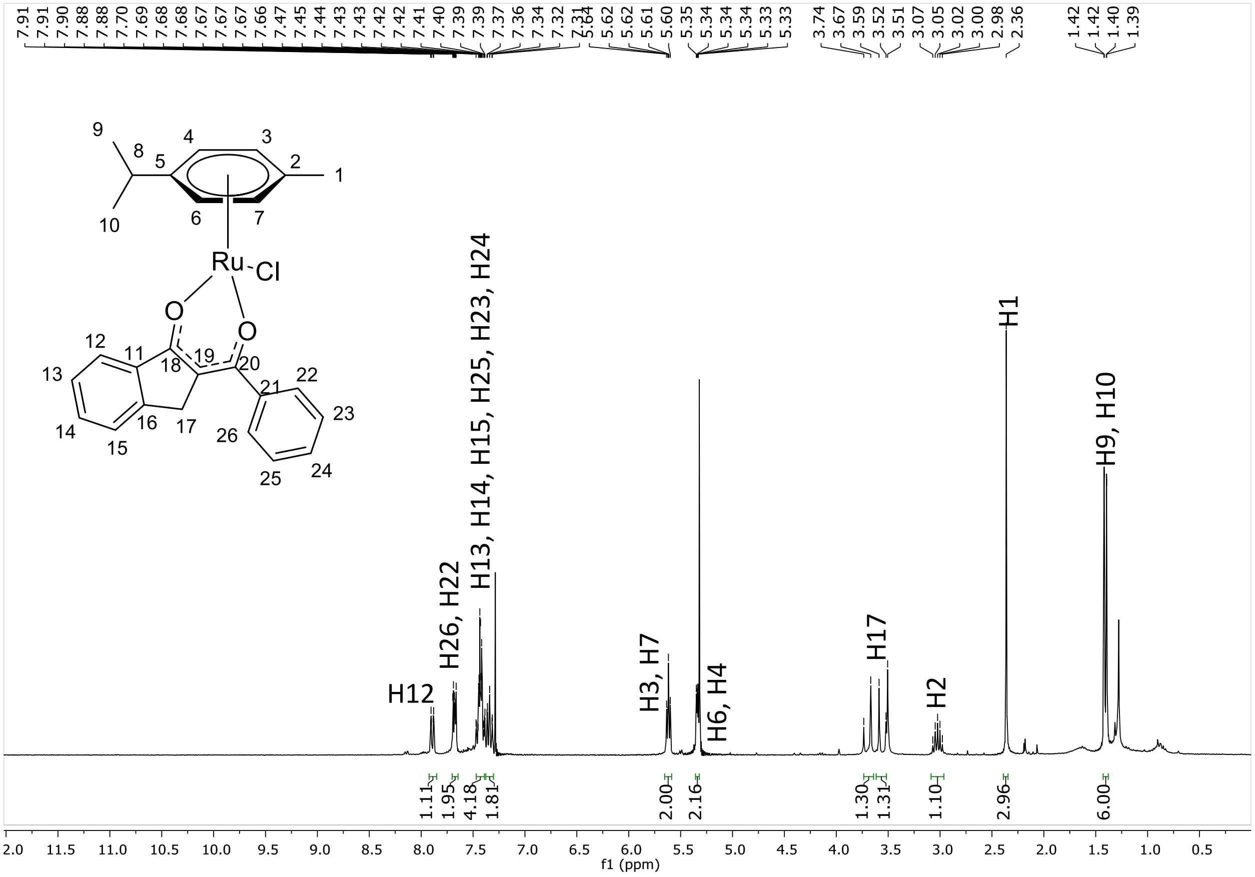

Figure S10: ^{13}C-NMR spectrum of **2a** in $CDCl_3$ 400 MHz:

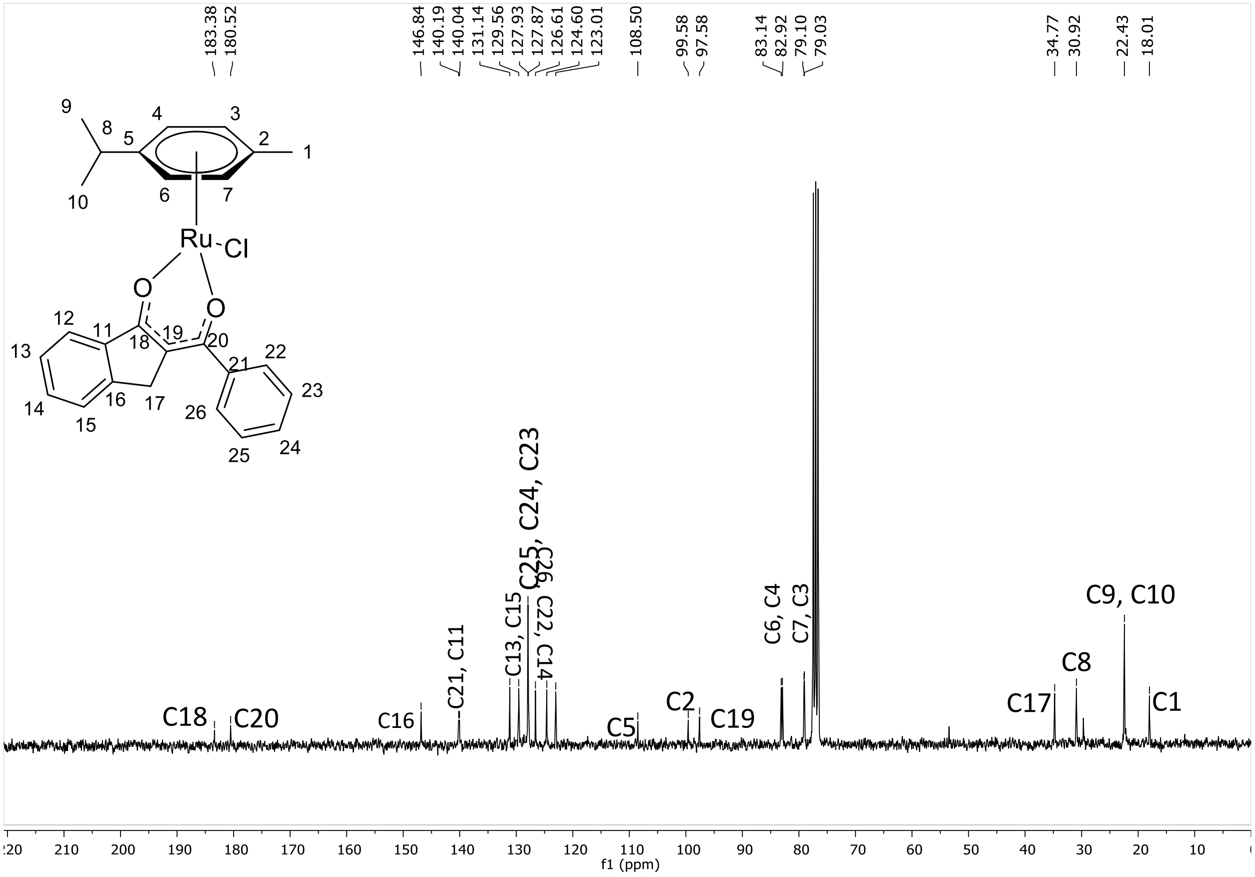

Figure S11: FTIR spectrum of complex **2b**.

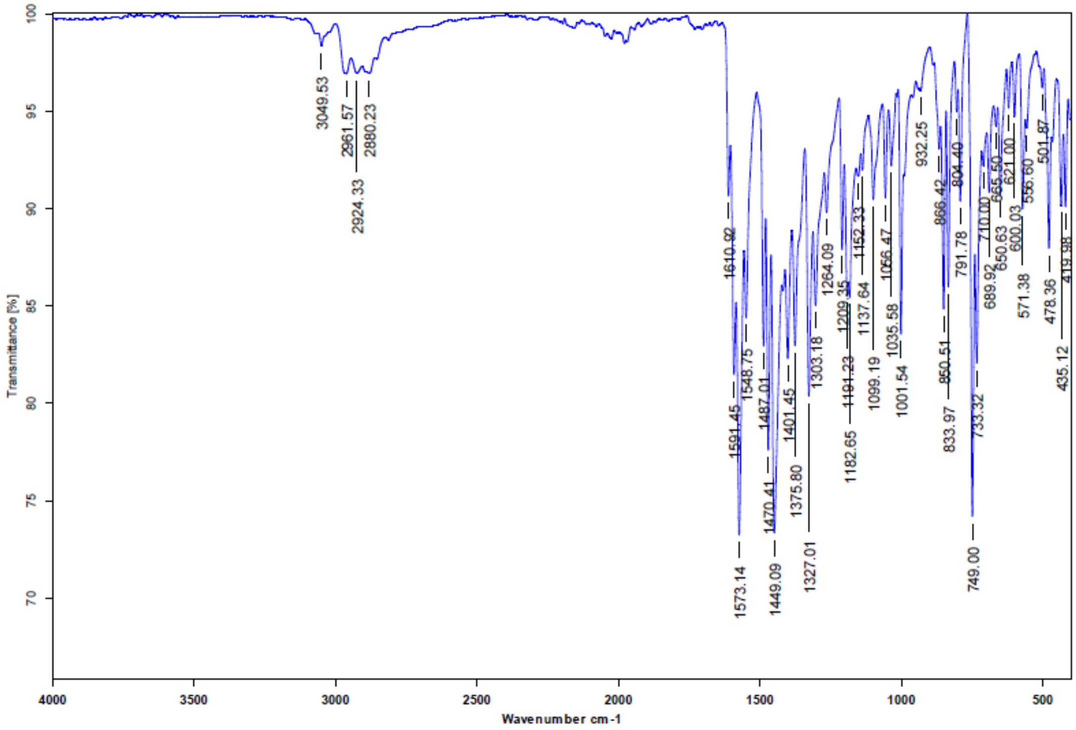

Figure S12: MS spectrum of complex **2b**.

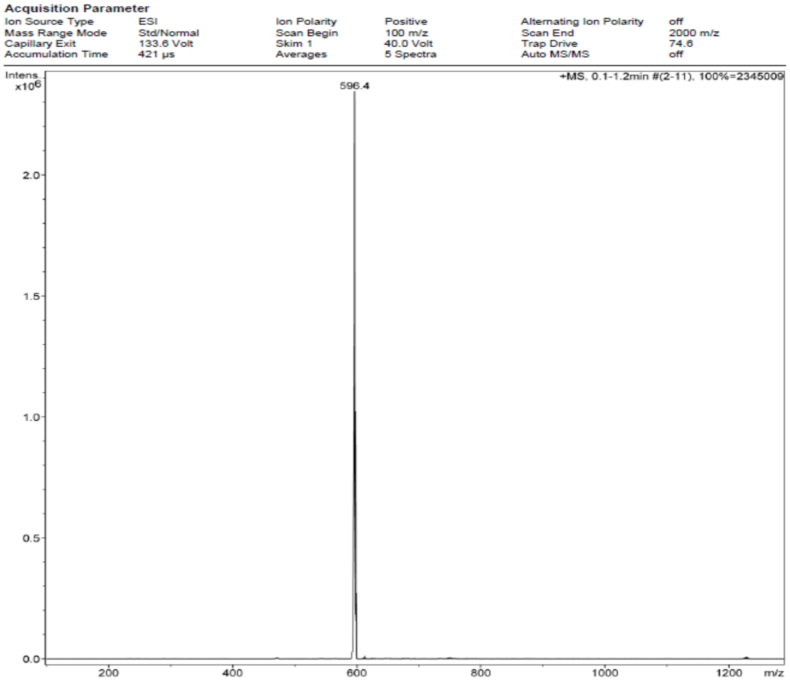

Figure S13: HRMS of complex **2b**.

**Sample Group**		**Info.**	
**Stream Name**	LC 1	**Acquisition SW Version**	6200 series TOF/6500 series Q-TOF B.06.01 (B6172 SP1)

User Spectra

**Fragmentor Voltage**	**Collision Energy**	**Ionization Mode**
150	0	ESI (+)

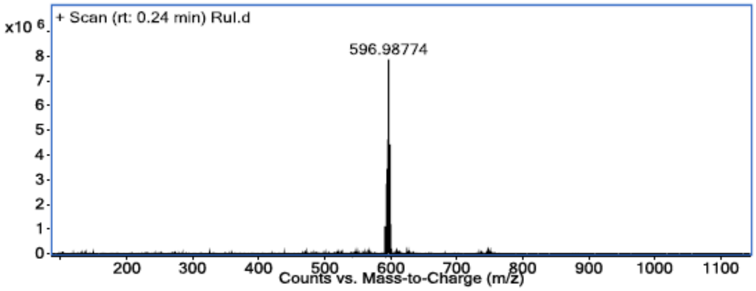

Peak List

m/z	z	Abund
590.99089	1	1136845.02
592.98899	1	456021.35
593.9888		2844940.35
594.98802		3505012.05
595.98877		4640274.74
596.98774	1	8072395.01
597.17802		736918.85
597.99047	1	2234367.25
598.9884	1	4444355.6
599.99222	1	1228913.79

Figure S14: ^{1}H-NMR spectrum of complex **2b** in $CDCl_3$ 400 MHz:

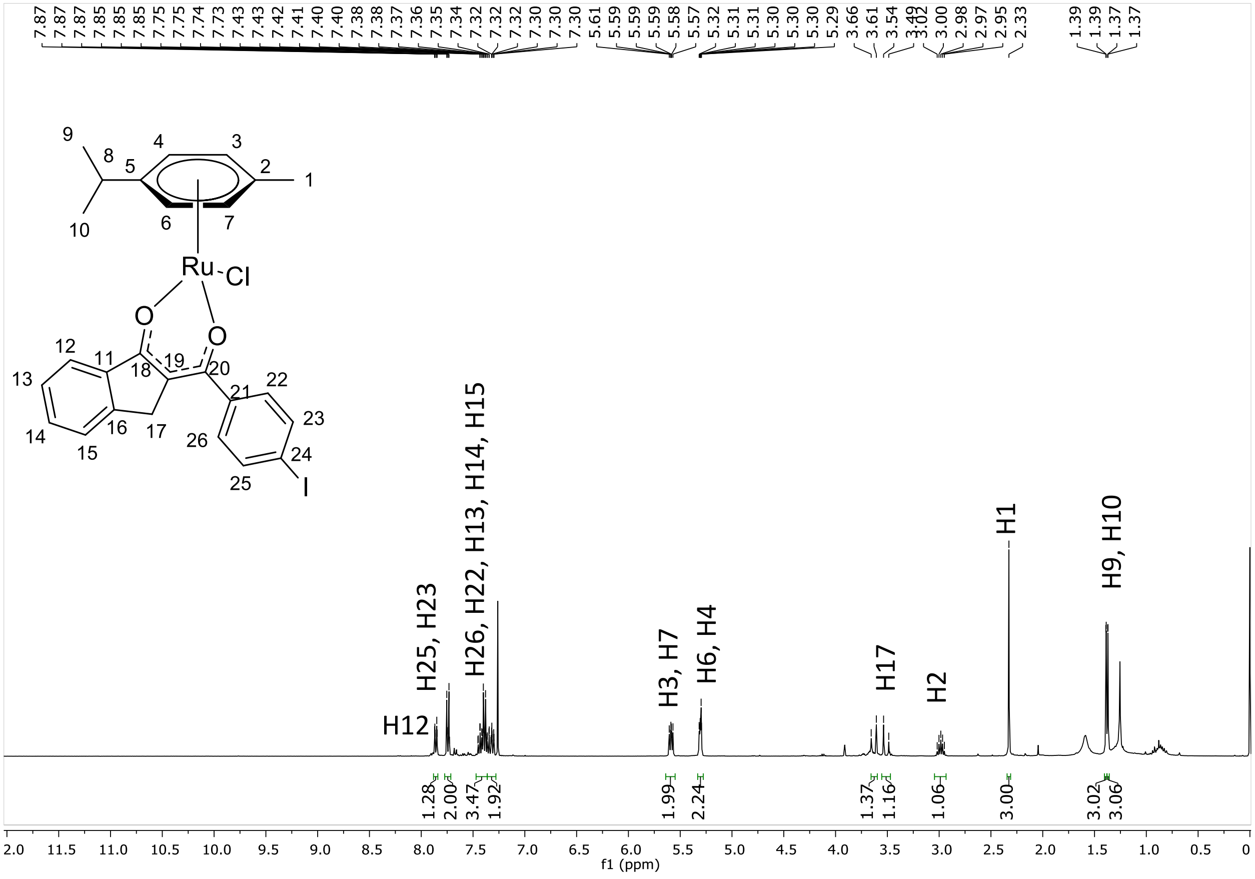

Figure S15: ^{13}C-NMR spectrum of complex **2b** in $CDCl_3$ 400 MHz:

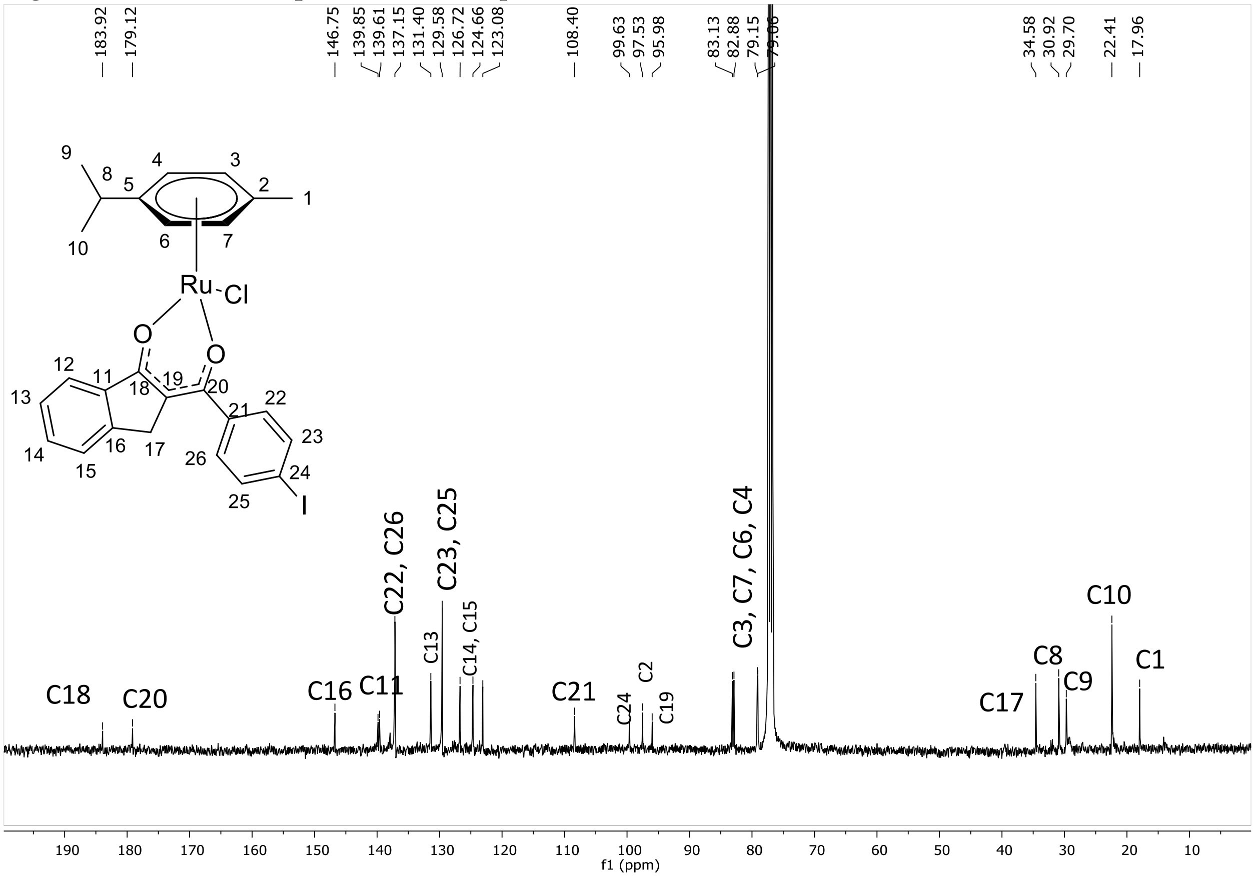

Figure S16: IR spectrum of complex **2c**.

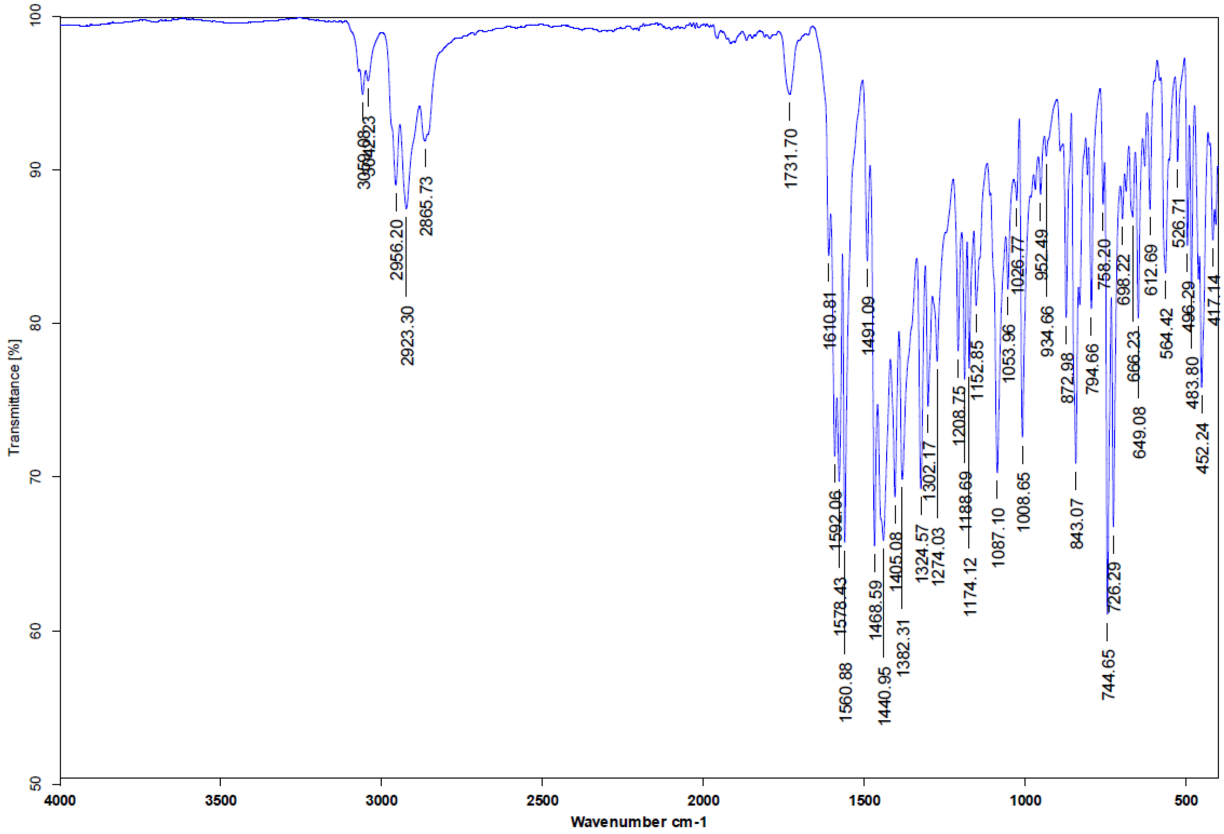

Figure S17: MS spectrum of complex **2c**.

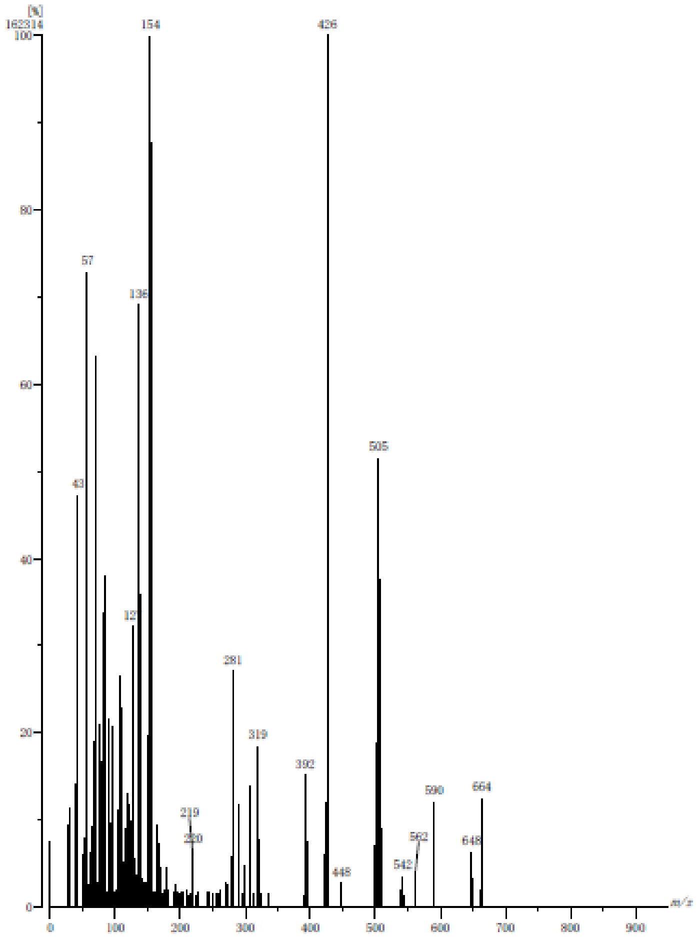

Figure S18: HRMS of complex **2c**.

Inlet : Direct Ion Mode : FAB+
RT : 1.58 min Scan# : (34,38)
Elements : C 26/0, H 30/0, Cl 2/0, O 2/0, Ru 1/0
Mass Tolerance : 1000ppm, 10mmu if m/z > 10
Unsaturation (U.S.) : 0.0 - 30.0

	Observed m/z	Int%							
	540.0201	4.00							
	Estimated m/z	Err[ppm / mmu]	U.S.	C	H	Cl	O	Ru
1	540.0197	+0.8 / +0.4	14.5	26	24	2	2	1

Figure S19: ^{1}H-NMR spectrum of **2c** in $CDCl_3$ 400 MHz:

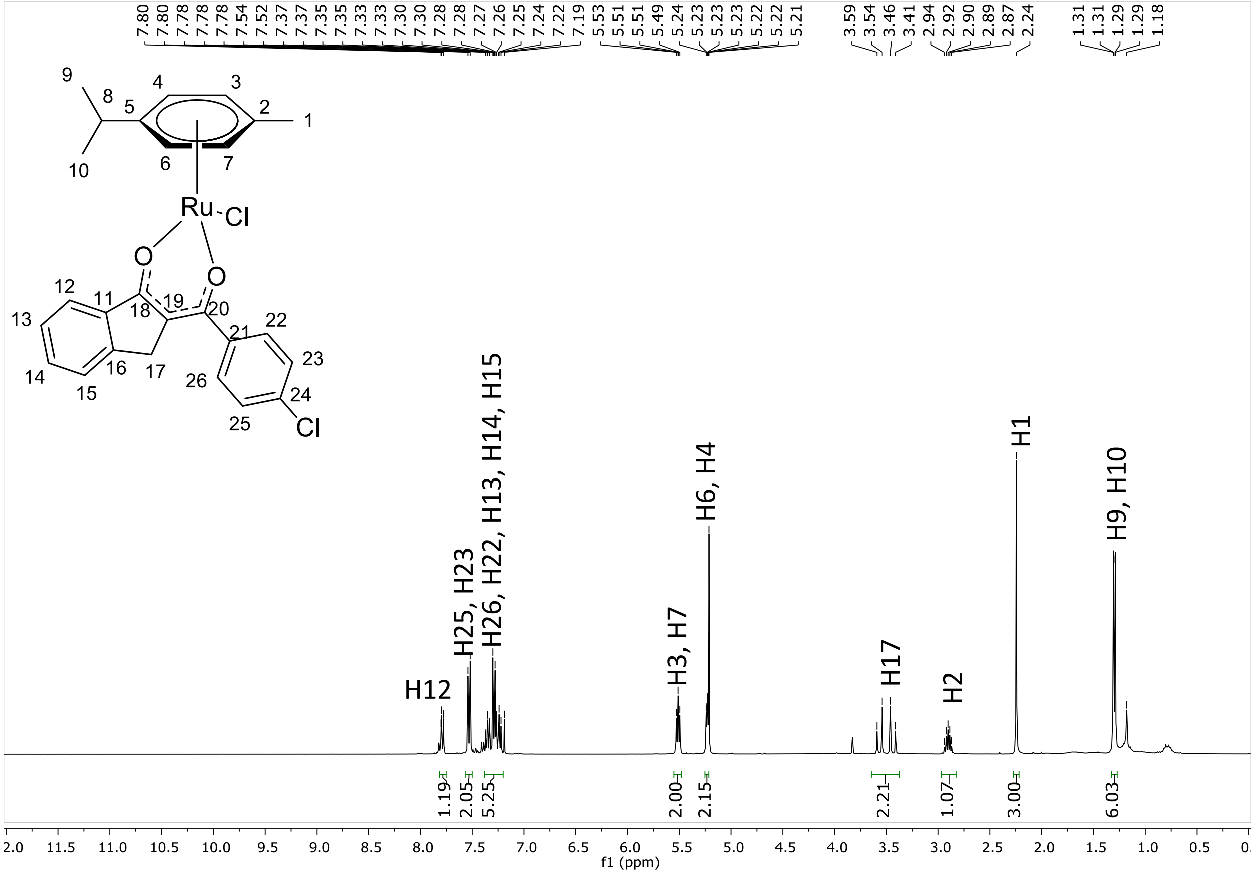

Figure S20: ^{13}C-NMR spectrum of **2c** in $CDCl_3$ 400 MHz:

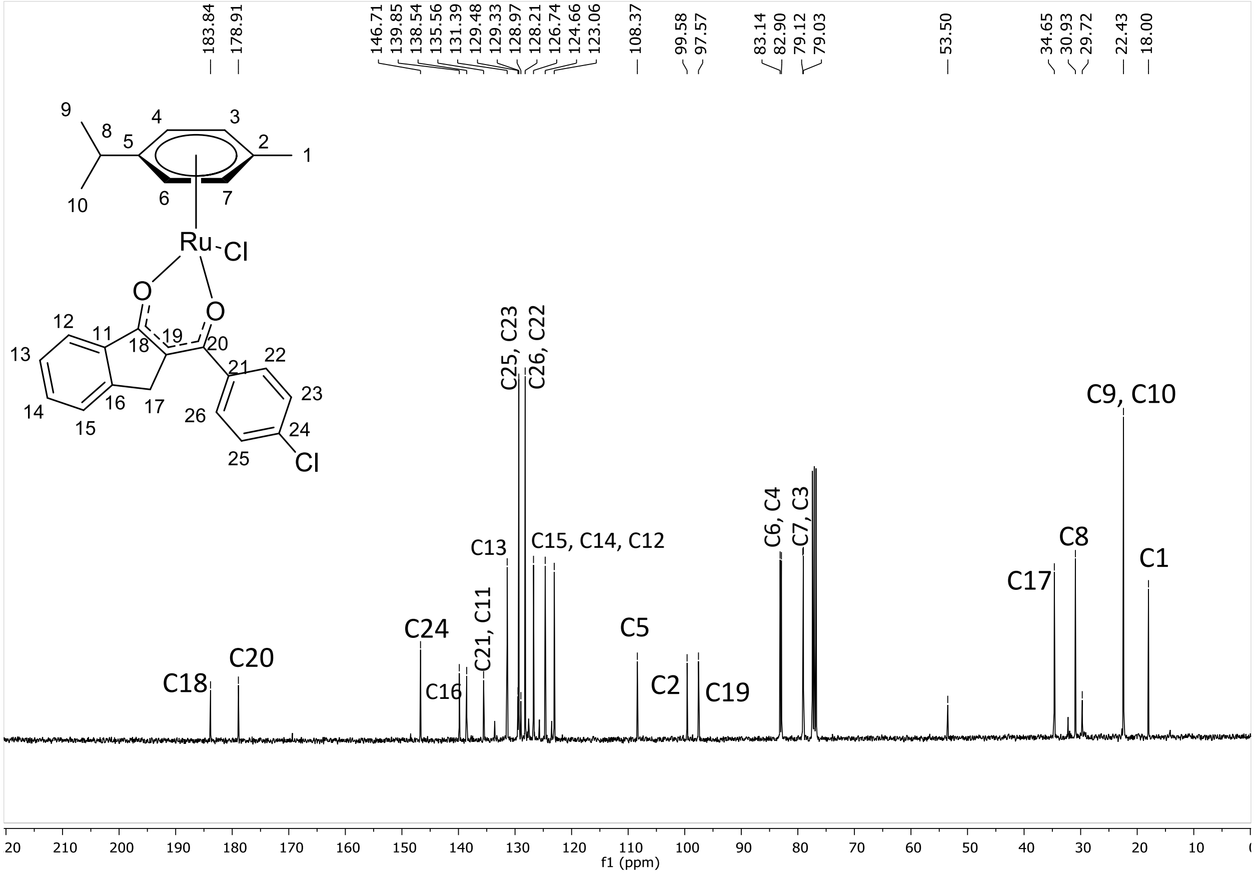

Figure S21: HSQC 2D spectrum of complex **2c**.

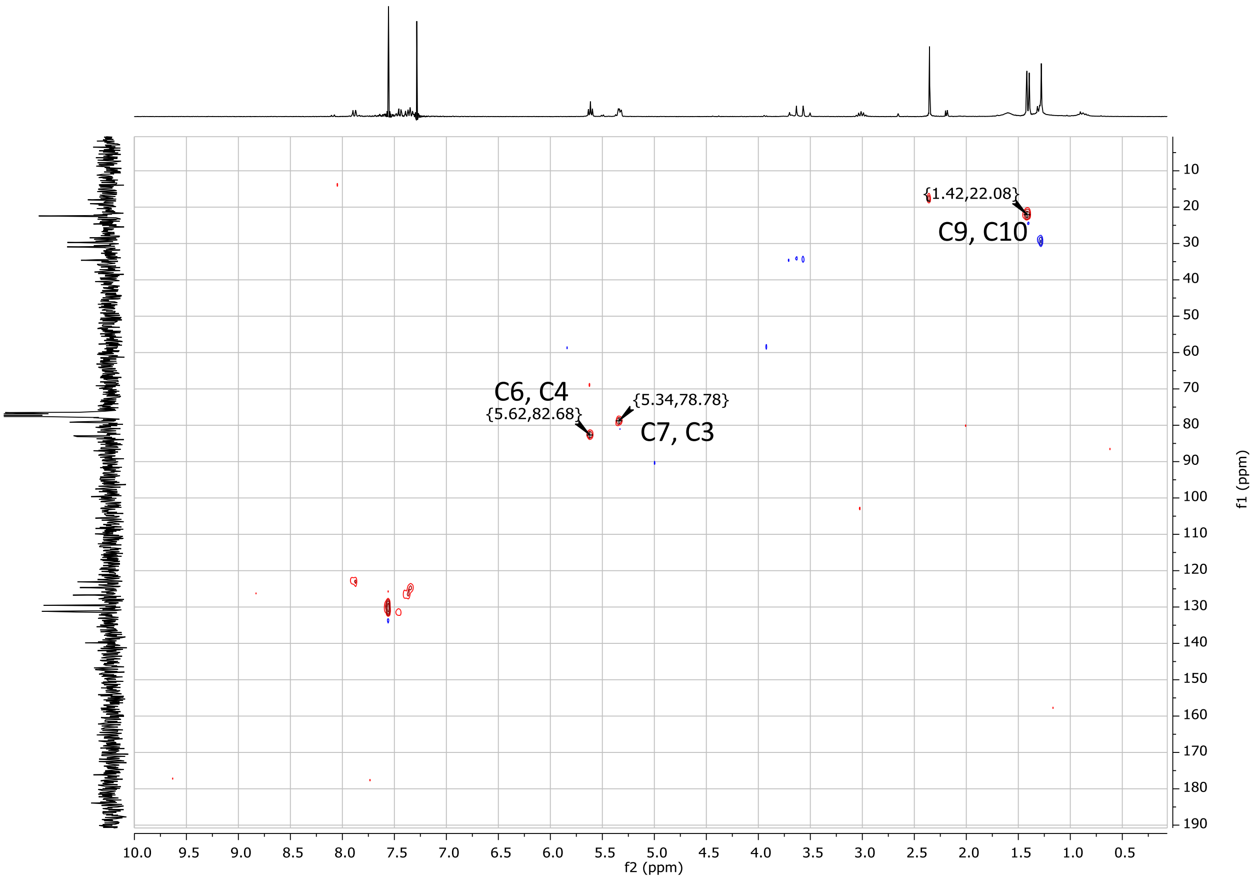

Figure S22: FTIR spectrum of complex **2d**.

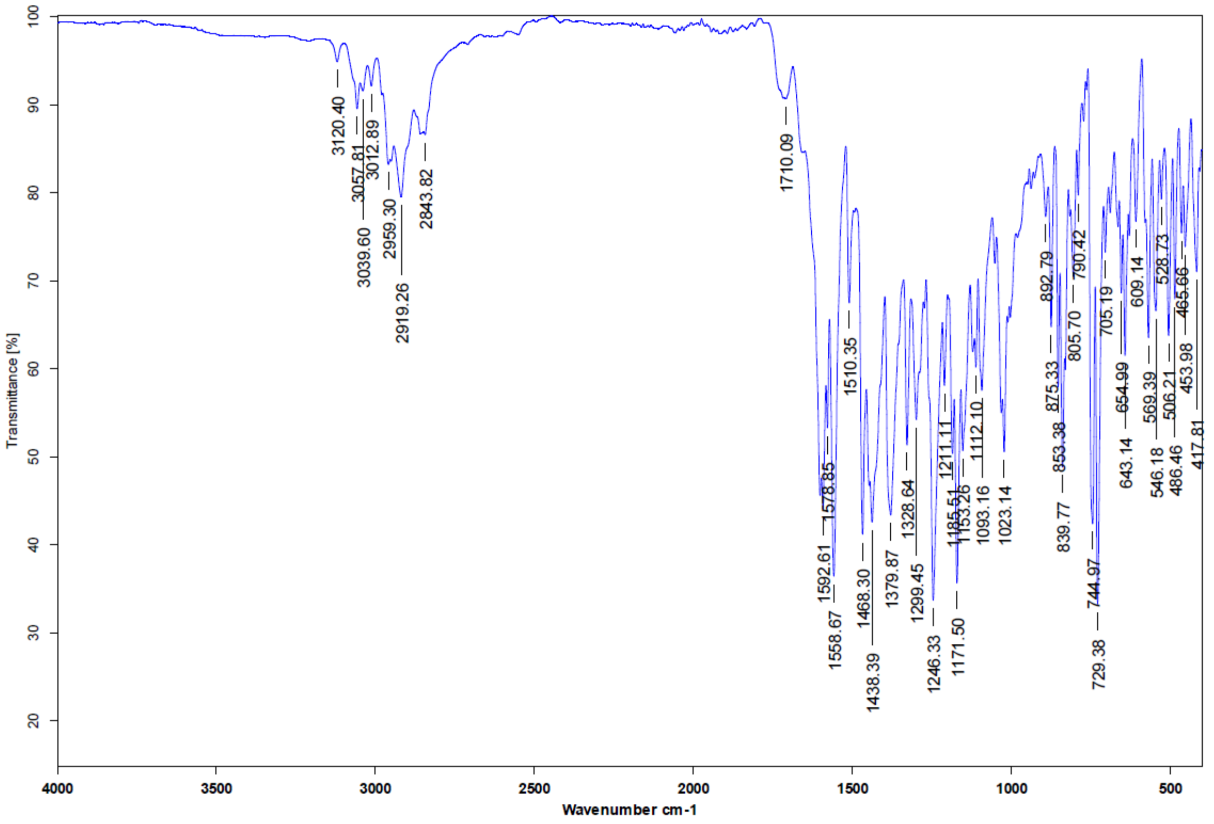

Figure S23: MS spectrum of complex **2d**.

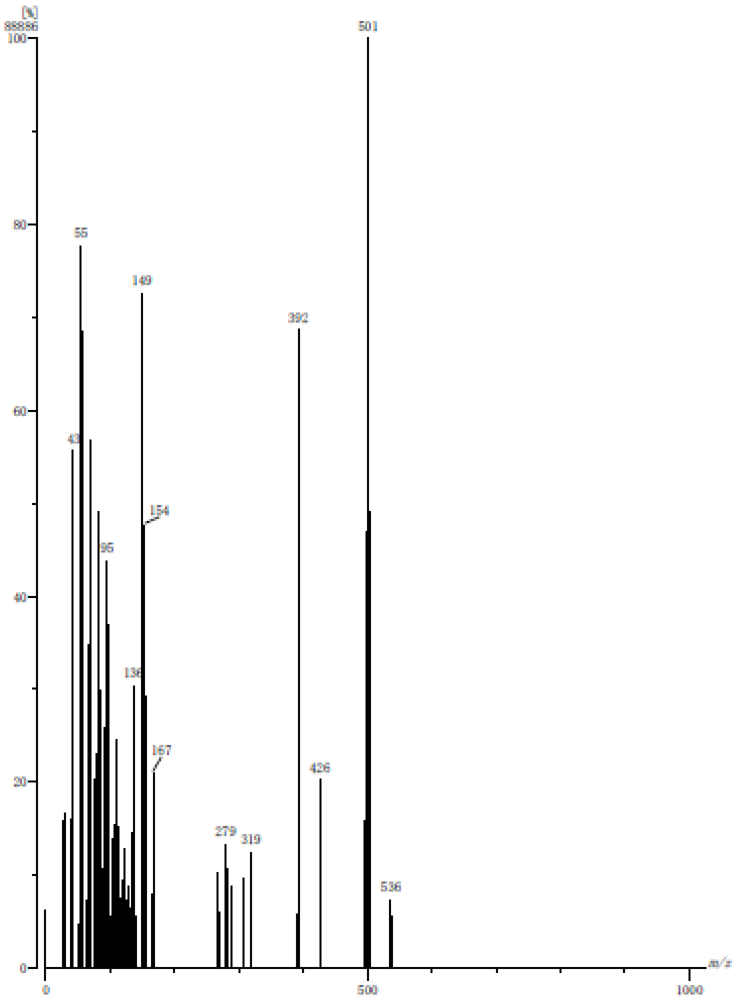

Figure S24: HRMS of complex **2d**.

Inlet : Direct Ion Mode : FAB+
RT : 0.19 min Scan# : (5,9)
Elements : C 27/0, H 30/0, Cl 1/0, O 3/0, Ru 1/0
Mass Tolerance : 1000ppm, 5mmu if m/z > 5
Unsaturation (U.S.) : 0.0 - 30.0

	Observed m/z	Int%
	536.0672	1.49

	Estimated m/z	Err[ppm / mmu]	U.S.	C	H	Cl	O	Ru
1	536.0692	-3.8 / -2.0	14.5	27	27	1	3	1

Figure S25: ^{1}H-NMR spectrum of complex **2d** in $CDCl_3$ 400 MHz:

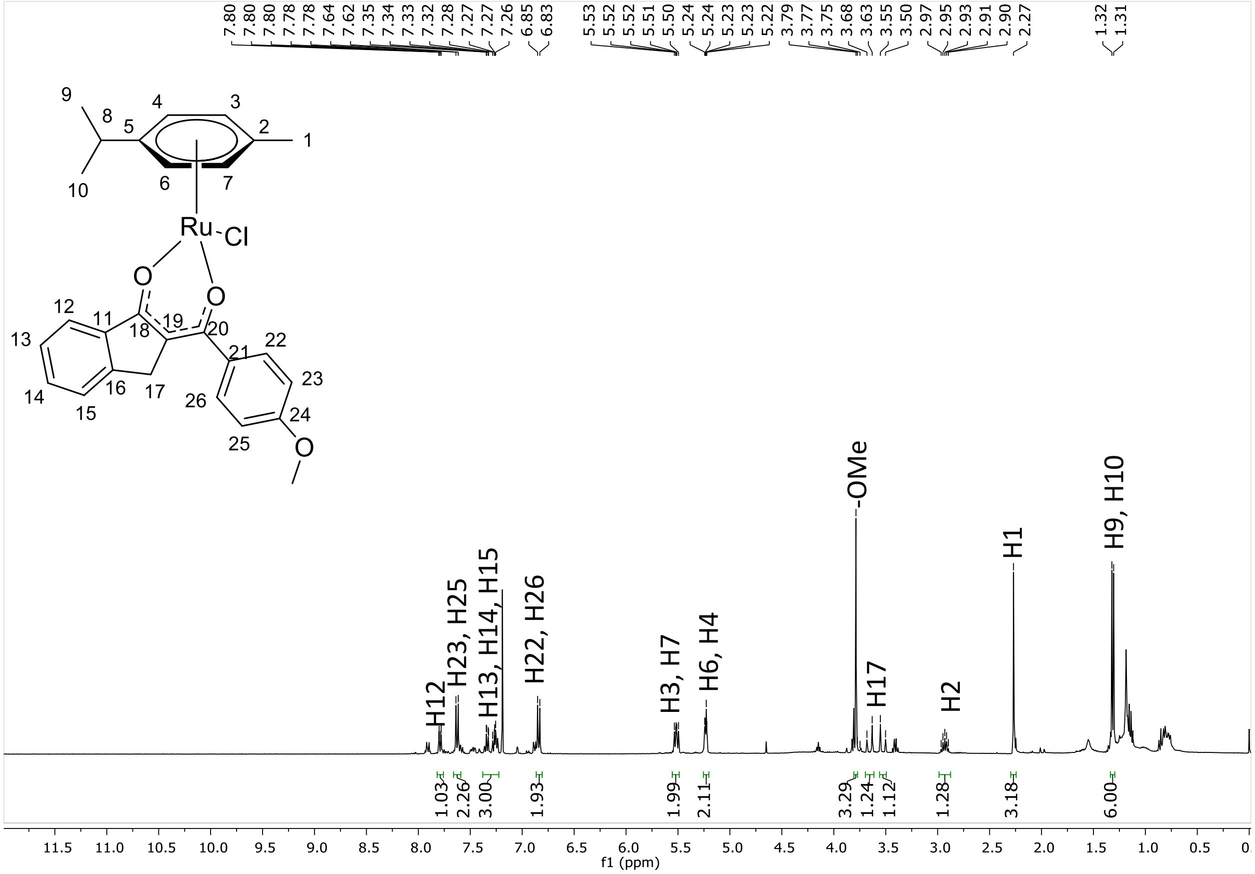

Figure S26: ^{13}C-NMR spectrum of **2d** in $CDCl_3$ 400 MHz:

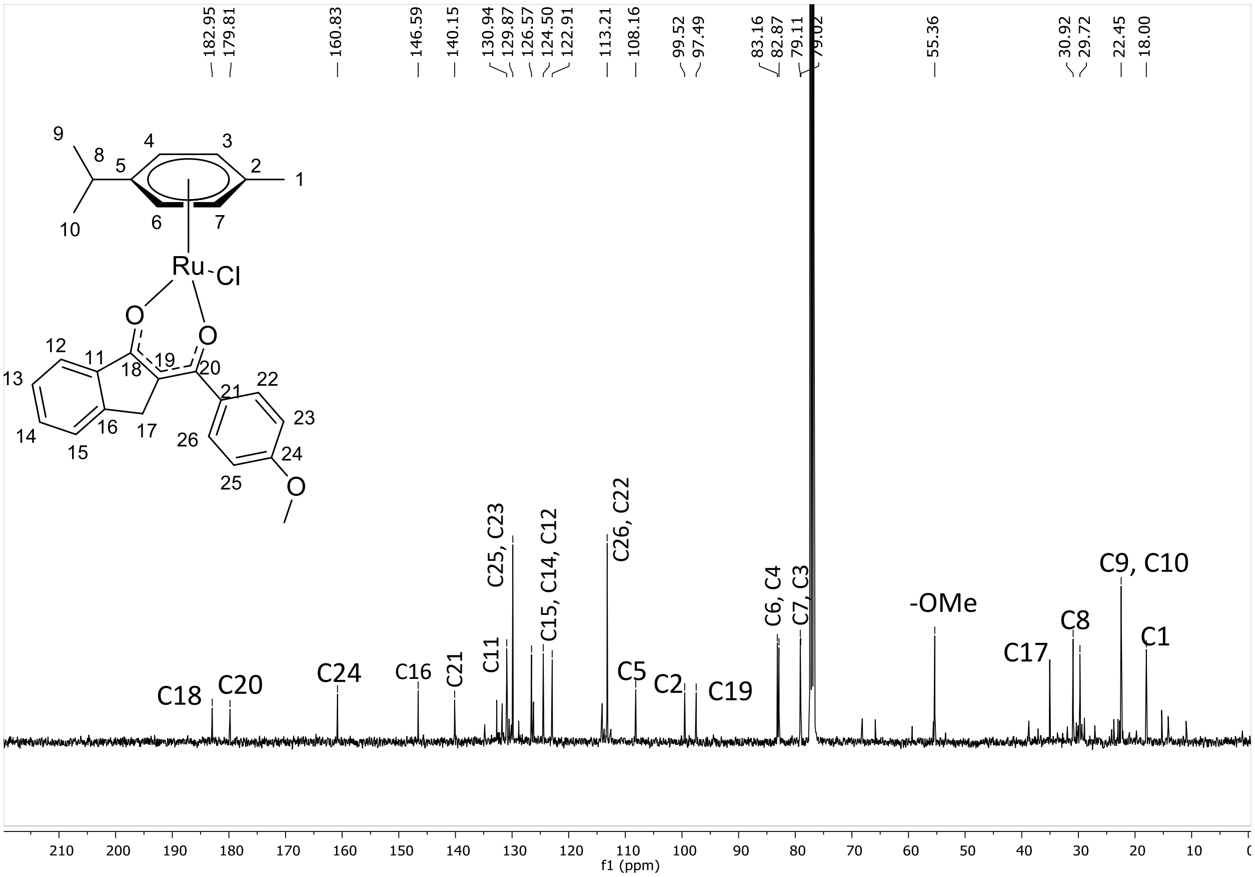

Figure S27: FTIR spectrum of complex **2e**.

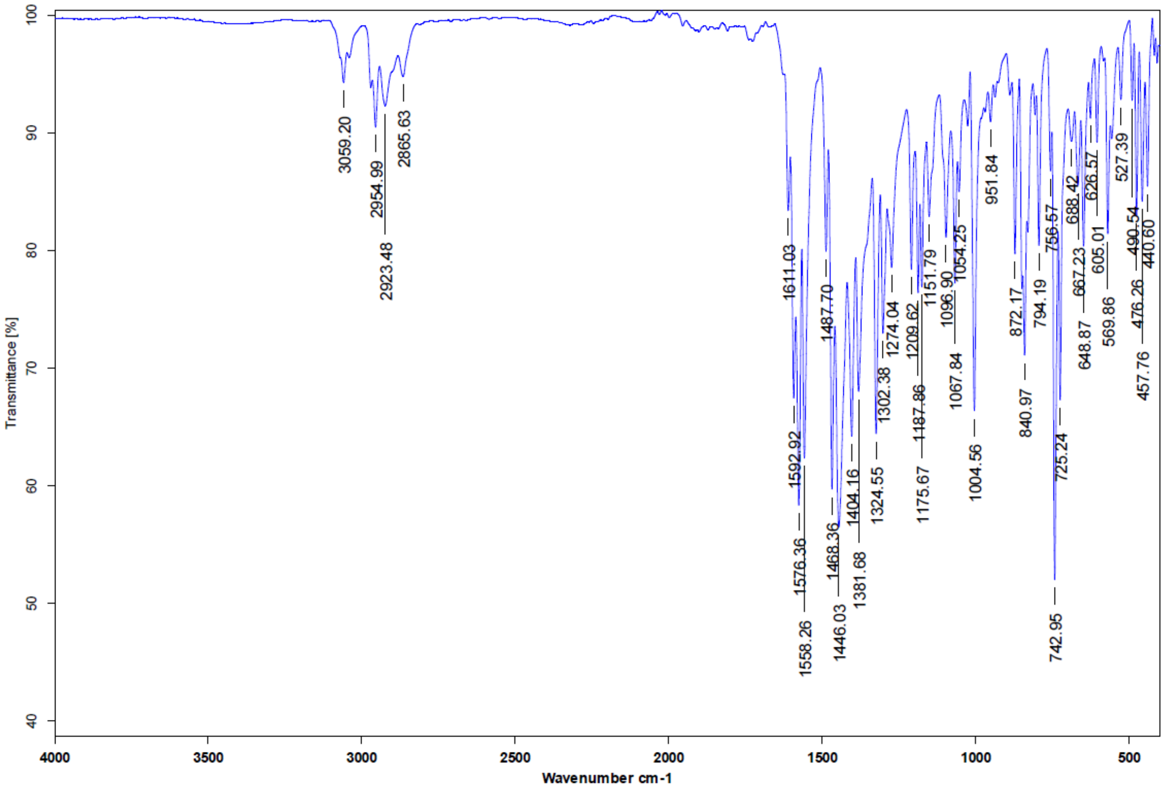

Figure S28: MS spectrum of complex **2d**.

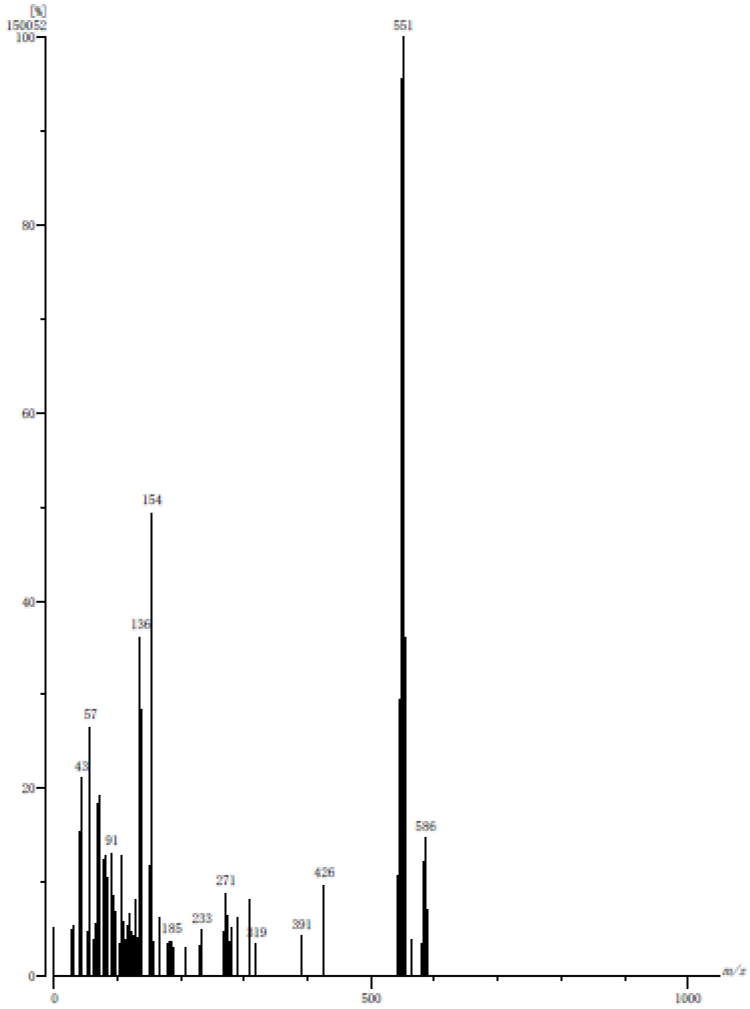

Figure S29: HRMS of complex **2e**.

Inlet : Direct   Ion Mode : FAB+
RT : 1.29 min   Scan# : (27,28)
Elements : C 26/0, H 26/0, Br 1/0, Cl 1/0, O 2/0, Ru 1/0
Mass Tolerance   : 1000ppm, 10mmu if m/z > 10
Unsaturation (U.S.) : 0.0 - 30.0

	Observed m/z	Int%							
	583.9691	2.90							
	Estimated m/z	Err[ppm / mmu]	U.S.	C	H	Br	Cl	O	Ru
1	583.9692	-0.1 / -0.1	14.5	26	24	1	1	2	1

Figure S30: ^{1}HNMR spectrum of complex **2e**.

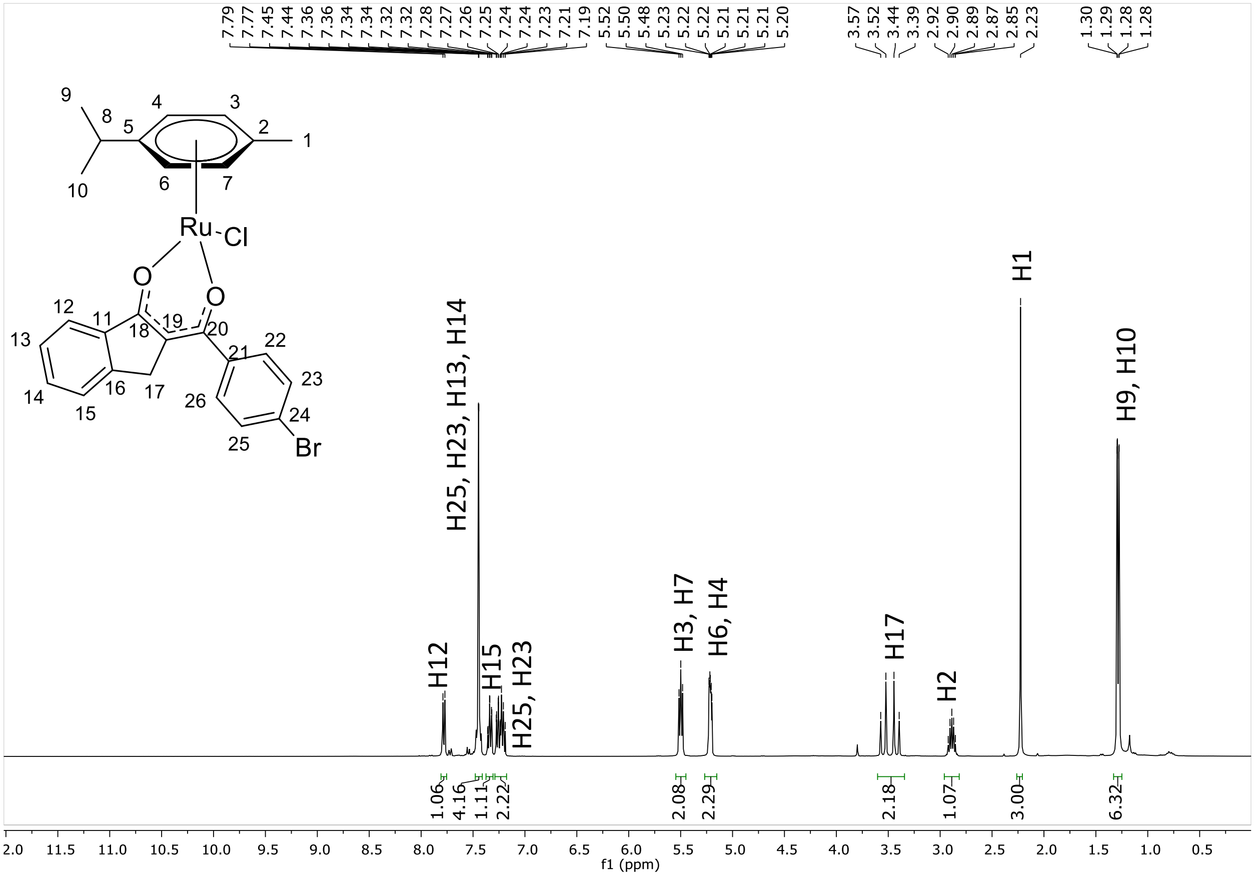

Figure S31: ^{13}CNMR spectrum of complex **2e**.

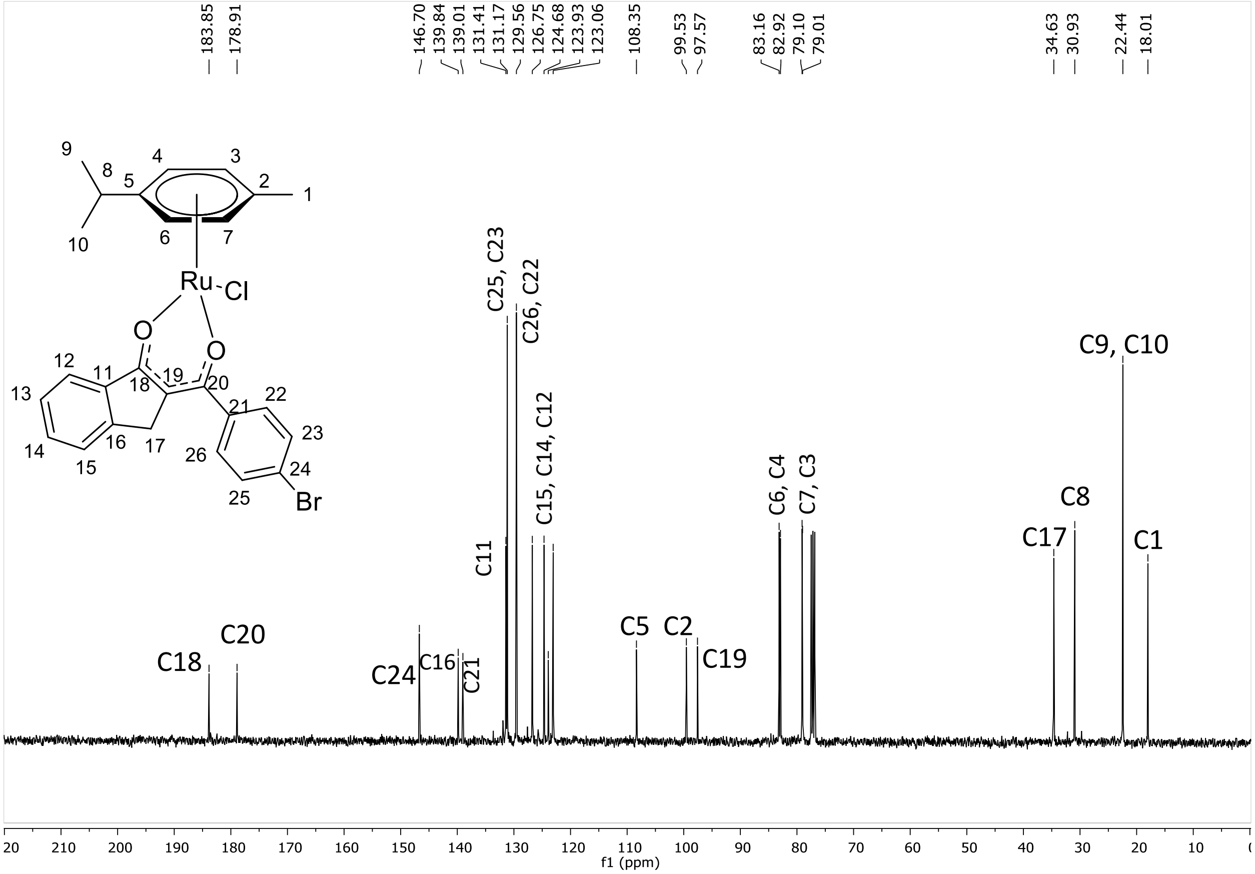

Figure S32: HSQC 2D spectrum of complex 2e in $CDCl_3$.

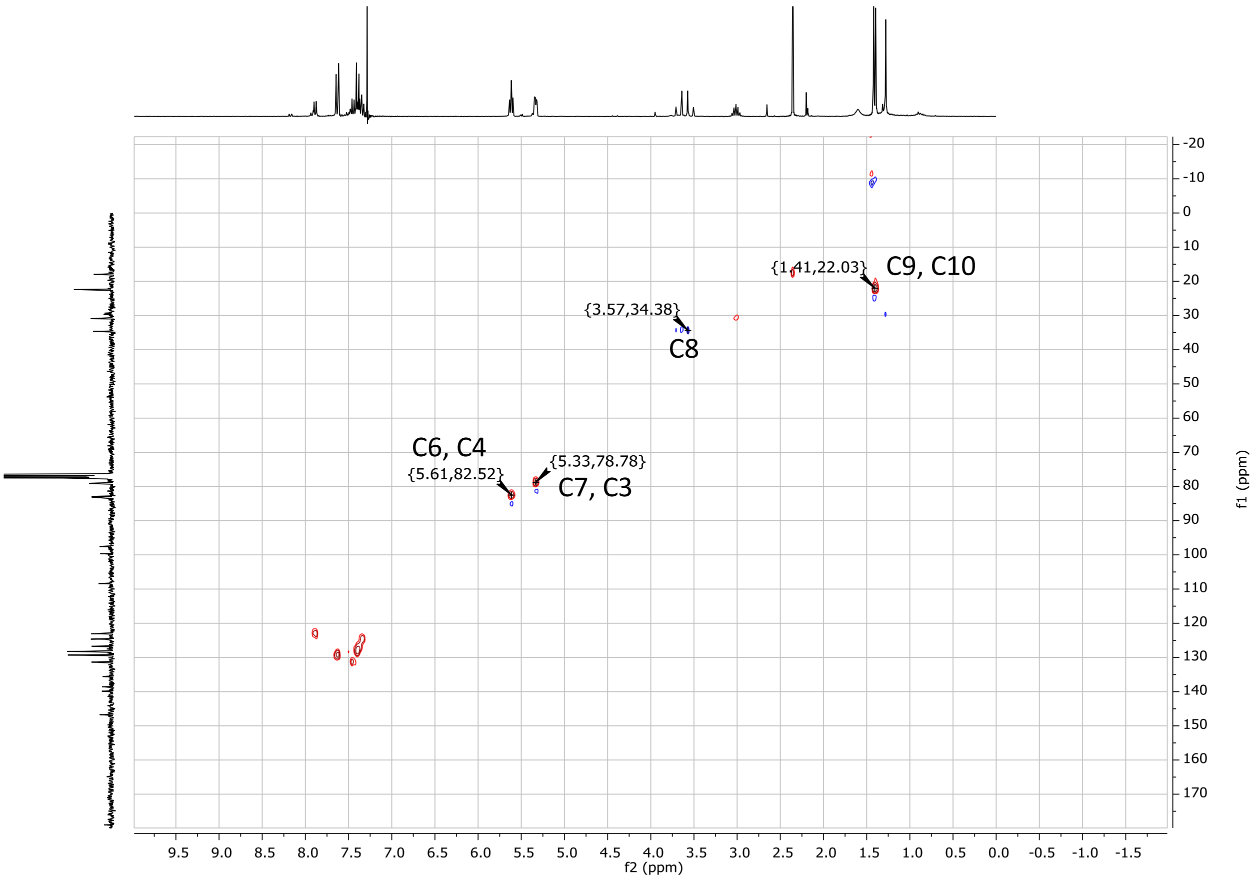

Synthesis of new ruthenium complexes and their exploratory study as organic semiconductors

Ricardo Ballinas-Indilí [1], María Elena Sánchez-Vergara [2,*], Saulo C. Rosales-Amezcua [3], Joaquín André Hernández Méndez [2], Byron Lópéz-Mayorga [4], René Miranda Ruvalcaba [1], and Cecilio Álvarez-Toledano [3]

[1] Departamento de Ciencias Químicas, Facultad de Estudios Superiores Cuautitlán Campo 1, Universidad Nacional Autónoma de México, Avenida 1o de Mayo s/n, Colonia Santa María las Torres, Cuautitlán Izcalli 54740, Mexico; mirruv@yahoo.com.mx (R.M.-R.)

[2] Facultad de Ingeniería, Universidad Anáhuac México, Av. Universidad Anáhuac 46, Col. Lomas Anáhuac, Huixquilucan 52786, Mexico

[3] Instituto de Química, Universidad Nacional Autónoma de México, Circuito Exterior s/n, Ciudad Universitaria, Mexico City 04510, Mexico; cecilio@unam.mx (C.Á.-T.)

[4] Escuela de Química, Facultad de Ciencias Químicas y Farmacia, Universidad de San Carlos de Guatemala, 11 avenida, Ciudad de Guatemala 01012, Guatemala, byron.lopez@profesor.usac.edu.gt (B.L.-M)

* Correspondence: elena.sanchez@anahuac.mx

Content

Complex **2a** (FTIR KBr and Hybrid Film ………………… IR1-IR2

Complex **2b** (FTIR KBr and Hybrid Film) ………………… IR3-IR4

Complex **2c** (FTIR KBr and Hybrid Film) ………………… IR5-IR6

Complex **2d** (FTIR KBr and Hybrid Film) ………………… IR7-IR8

Complex **2e** (FTIR KBr and Hybrid Film) ………………….. IR9-IR10

Figure IR1: FTIR spectrum of complex **2a** (KBr).

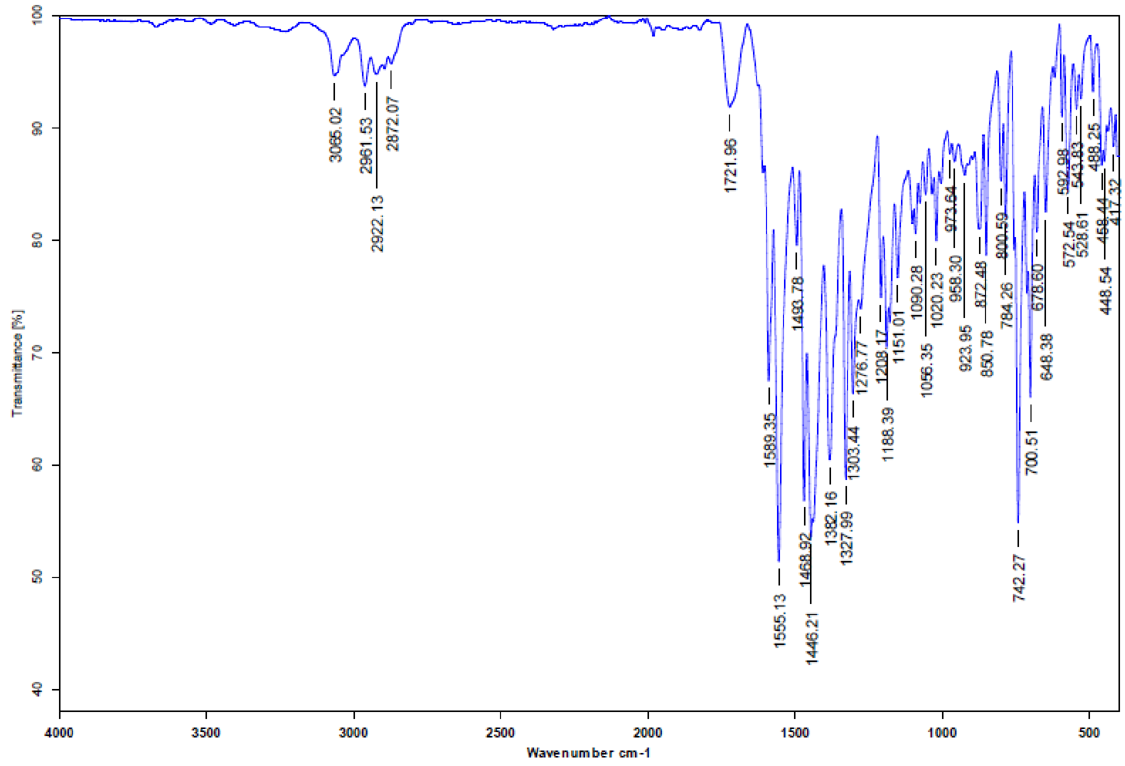

Figure IR2: FTIR spectrum of complex **2a** (hybrid film).

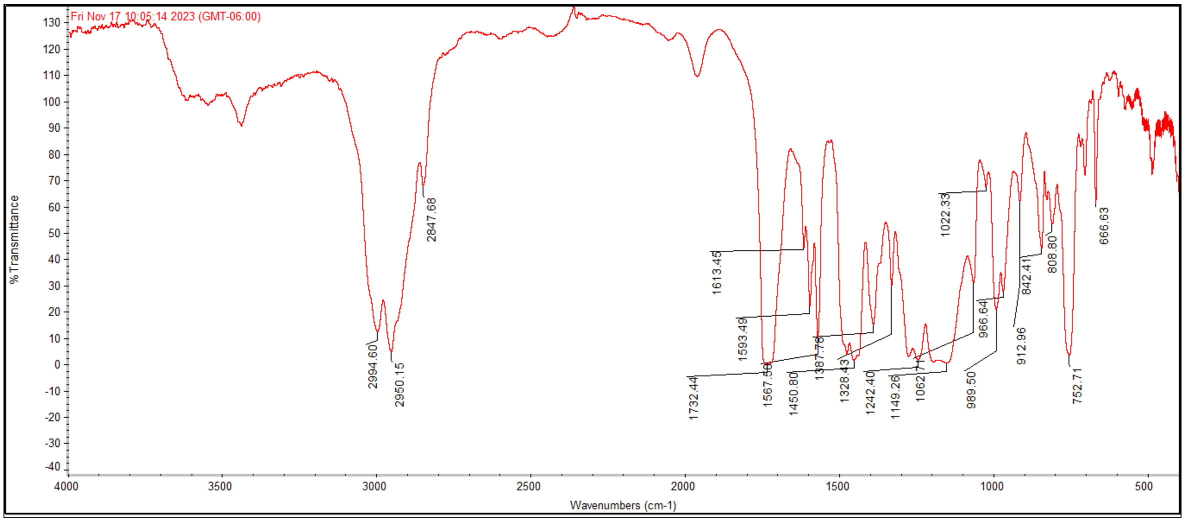

Figure IR3: FTIR spectrum of complex **2b** (KBr).

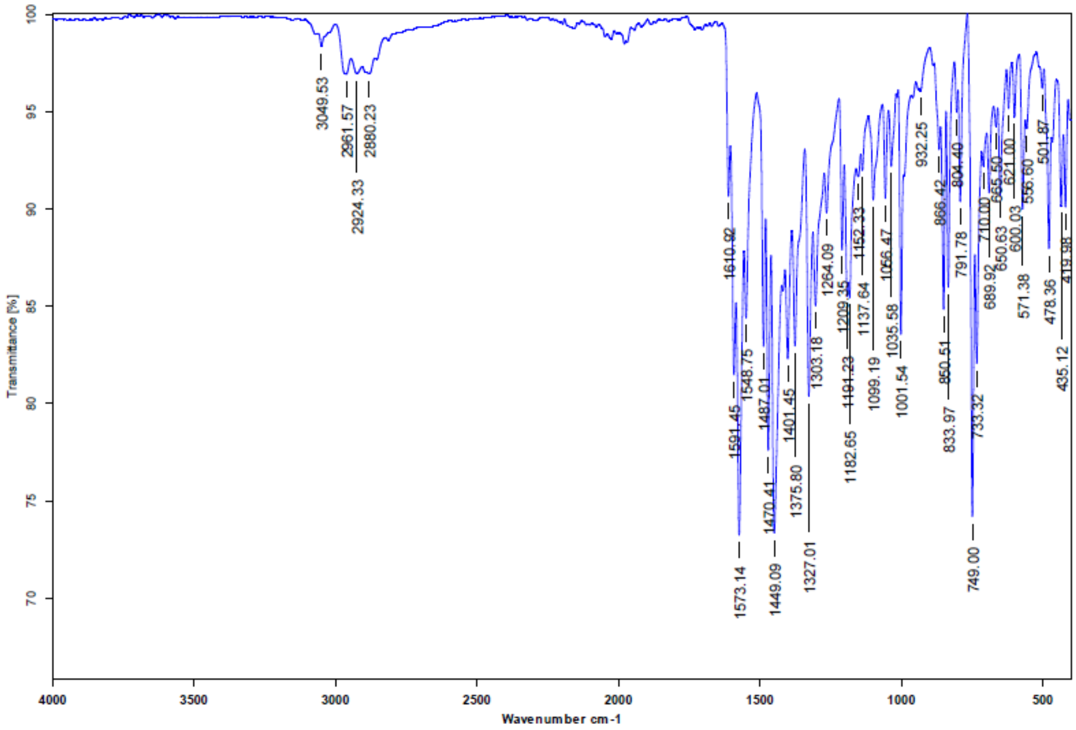

Figure IR4: FTIR spectrum of complex **2b** (hybrid film).

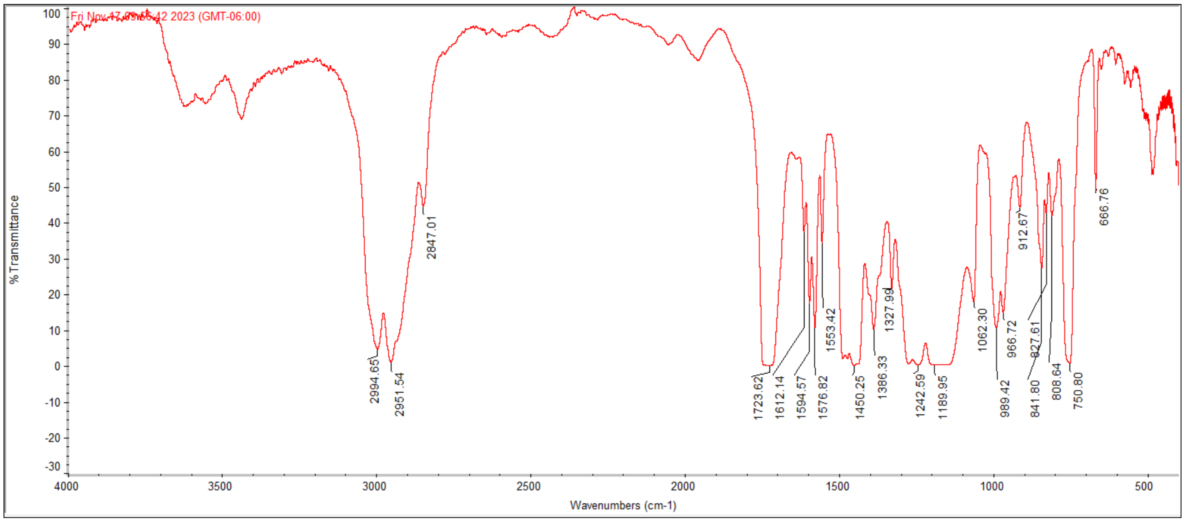

Figure IR5: IR spectrum of complex **2c** (KBr).

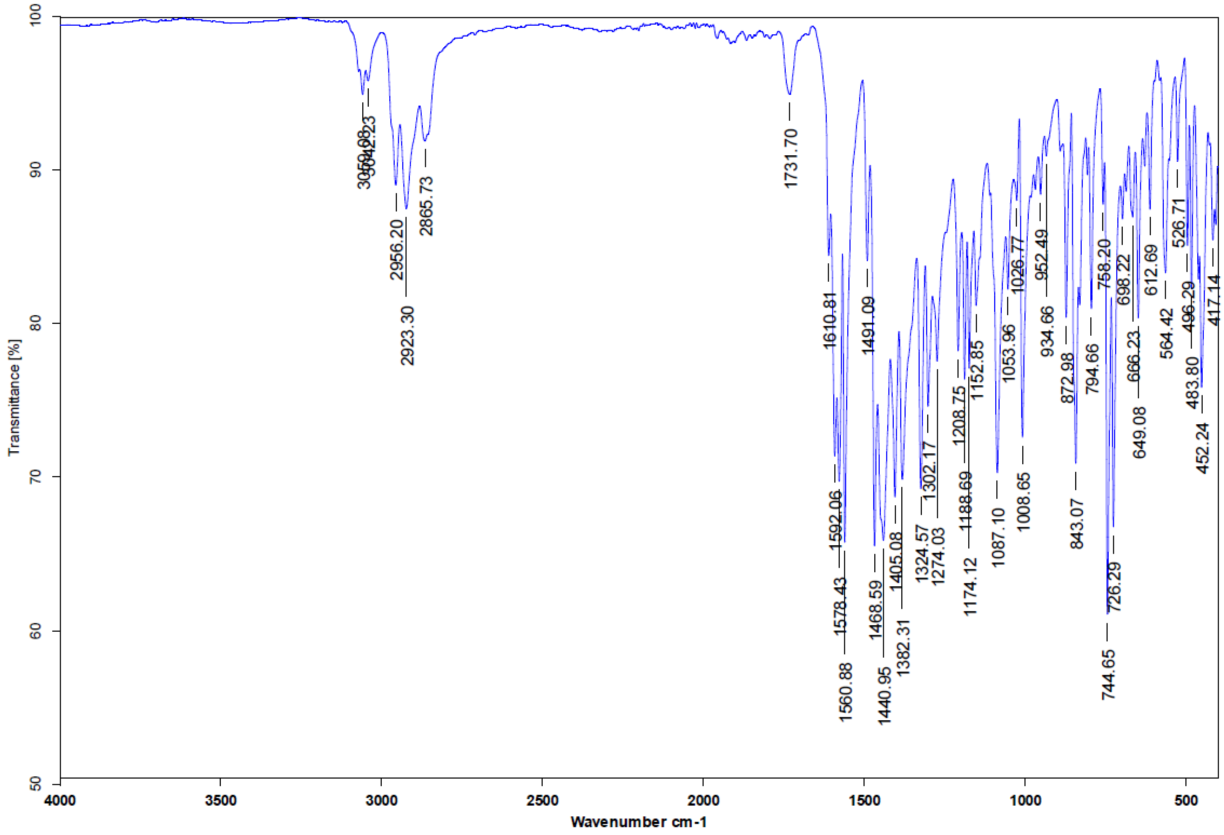

Figure IR6: FTIR spectrum of complex **2c** (hybrid film).

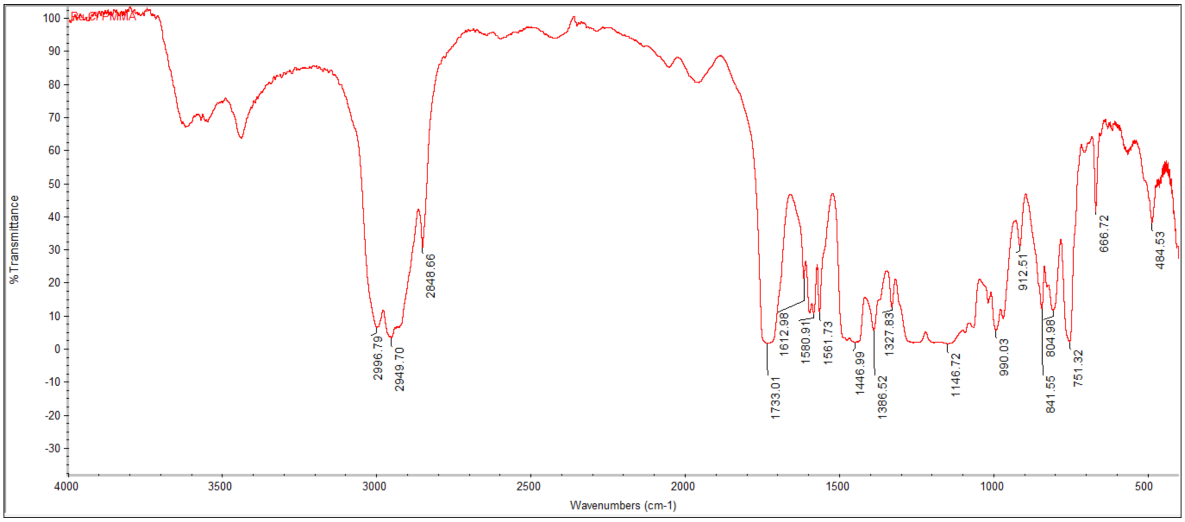

Figure IR7: FTIR spectrum of complex **2d** (KBr).

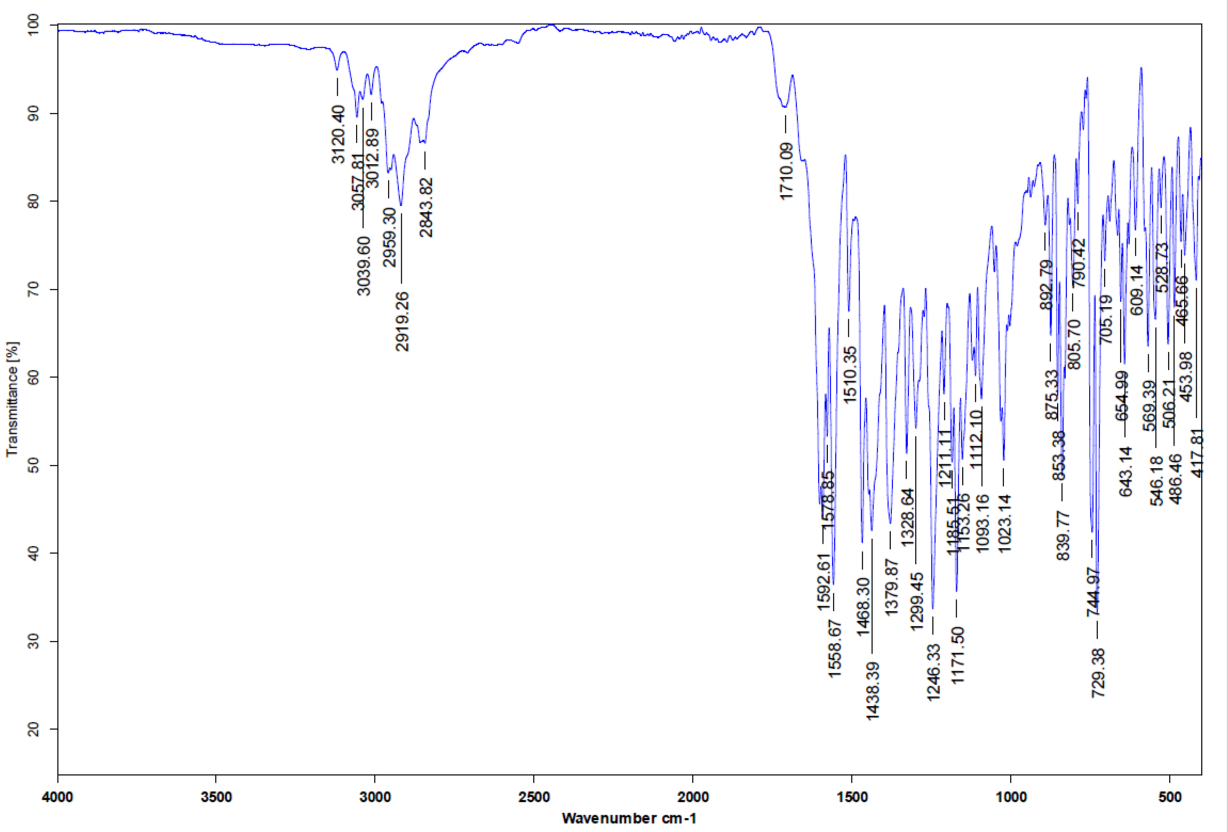

Figure IR8: FTIR spectrum of complex **2d** (hybrid film).

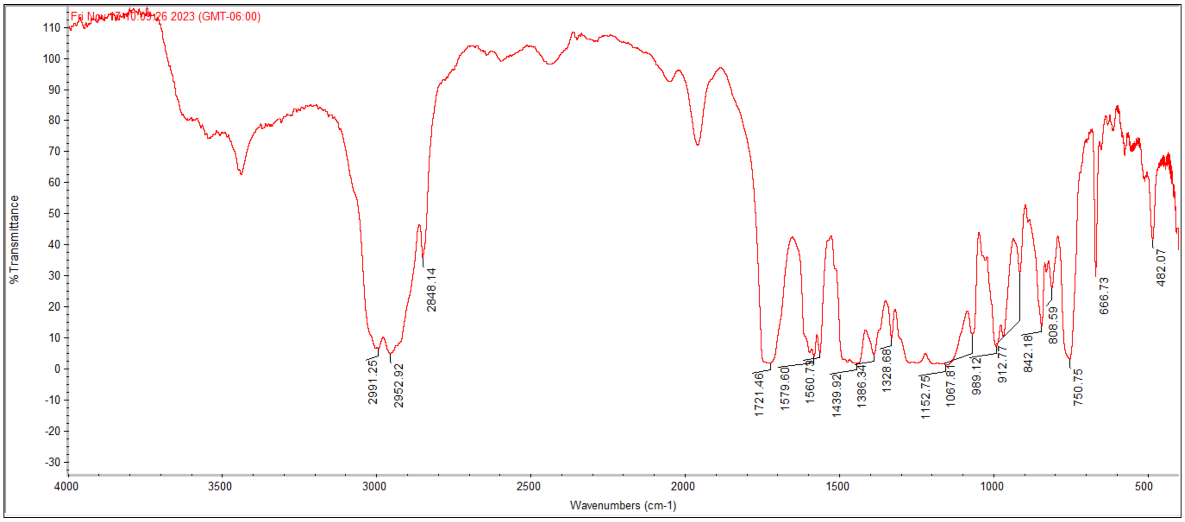

Figure IR9: FTIR spectrum of complex **2e** (KBr).

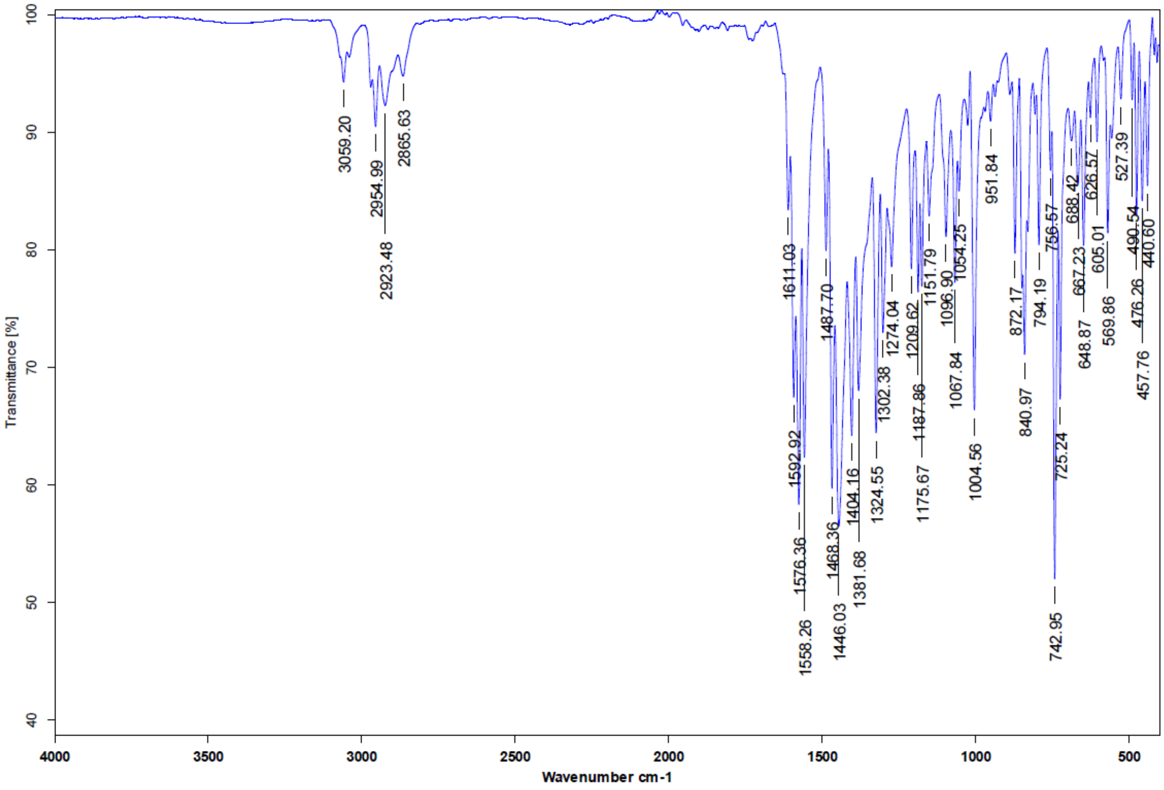

Figure IR10: FTIR spectrum of complex **2e** (hybrid film).

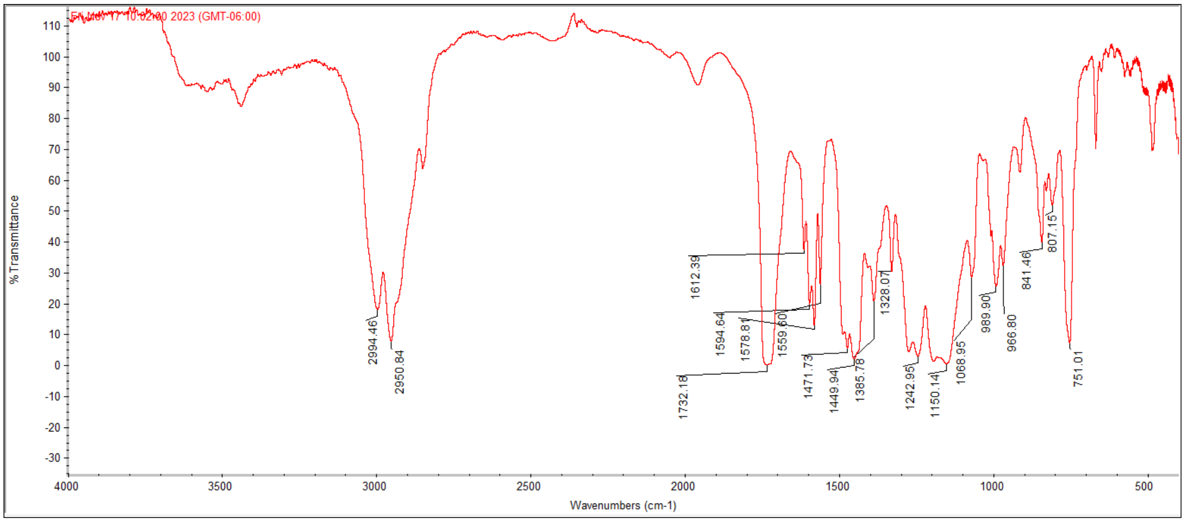

Supplement: Supplementary file 1 [file polymers-16-01338-s001.zip › polymers-2976926-supplementary.pdf]
